# Dominance hierarchy regulates social behavior during spatial movement

**DOI:** 10.3389/fnins.2024.1237748

**Published:** 2024-02-07

**Authors:** Ariel Lara-Vasquez, Nelson Espinosa, Cristian Morales, Constanza Moran, Pablo Billeke, Joseph Gallagher, Joshua J. Strohl, Patricio T. Huerta, Pablo Fuentealba

**Affiliations:** ^1^Centro Integrativo de Neurociencias y Departamento de Psiquiatría, Pontificia Universidad Católica de Chile, Santiago, Chile; ^2^Laboratorio de Neurociencia Social y Neuromodulación, Centro de Investigación en Complejidad Social, Universidad del Desarrollo, Santiago, Chile; ^3^Laboratory of Immune & Neural Networks, Feinstein Institutes for Medical Research, Manhasset, NY, United States; ^4^Department of Molecular Medicine, Zucker School of Medicine at Hofstra/Northwell, Manhasset, NY, United States; ^5^Centro de Investigación en Nanotecnología y Materiales Avanzados – CIEN-UC, Pontificia Universidad Católica de Chile, Santiago, Chile

**Keywords:** dominance hierarchy, spatial navigation, prefrontal cortex, hippocampus, cortical oscillations, social behavior, medial prefrontal cortex

## Abstract

Rodents establish dominance hierarchy as a social ranking system in which one subject acts as dominant over all the other subordinate individuals. Dominance hierarchy regulates food access and mating opportunities, but little is known about its significance in other social behaviors, for instance during collective navigation for foraging or migration. Here, we implemented a simplified goal-directed spatial task in mice, in which animals navigated individually or collectively with their littermates foraging for food. We compared between conditions and found that the social condition exerts significant influence on individual displacement patterns, even when efficient navigation rules leading to reward had been previously learned. Thus, movement patterns and consequent task performance were strongly dependent on contingent social interactions arising during collective displacement, yet their influence on individual behavior was determined by dominance hierarchy. Dominant animals did not behave as leaders during collective displacement; conversely, they were most sensitive to the social environment adjusting their performance accordingly. Social ranking in turn was associated with specific spontaneous neural activity patterns in the prefrontal cortex and hippocampus, with dominant mice showing higher firing rates, larger ripple oscillations, and stronger neuronal entrainment by ripples than subordinate animals. Moreover, dominant animals selectively increased their cortical spiking activity during collective movement, while subordinate mice did not modify their firing rates, consistent with dominant animals being more sensitive to the social context. These results suggest that dominance hierarchy influences behavioral performance during contingent social interactions, likely supported by the coordinated activity in the hippocampal-prefrontal circuit.

## Introduction

Social behavior is an adaptive response that has evolved to improve ecological fitness in many species (Insel and Fernald, 2004). Mammalian social behaviors occur in the context of extended groups; however, in laboratory settings, interactions such as fighting, chasing, courtship, and grooming, are typically investigated in pairs of individuals (de Chaumont et al., 2012; Shemesh et al., 2013). This approach of studying dyads, and treating the results as prototypical social behavior, has significant limitations because animal groups commonly rely on more complicated social structures. Indeed, recent experiments tracking mice in ethologically relevant environments have revealed strongly correlated social behaviors that become evident in settings of not just two but multiple individuals (de Chaumont et al., 2012; Shemesh et al., 2013). Moreover, some social behaviors that arise in groups can be contingent, as occurs when individuals randomly meet during environmental exploration, or when they court a mating partner (Conradt and Roper, 2003). Conversely, social interactions can be a constitutive group property, such as the social ranking system (Lindzey et al., 1961). Dominance status in a social group can be important as it regulates individual behavior in assays evaluating anxiety, locomotion, or aggressiveness, to an extent comparable to genetic mutations or pharmacological agents (Lathe, 2004; Park et al., 2018). Nevertheless, most reports describe the role of dominance status only under individual conditions.

Previous studies have established that the neural basis of dominance hierarchy relies on the efficacy of synaptic transmission in the medial prefrontal cortex (mPFC) (Anacker et al., 2019), particularly that of pyramidal cells (Wang et al., 2011). Furthermore, synaptic activity in the mPFC as well as its functional connectivity with the dorsal hippocampus are essential to regulate spatial navigation and decision-making (Euston et al., 2012). More generally, it has been proposed that the mPFC processes the current context and compares it with past experience to predict and execute the most adaptive behavioral response (Matsumoto et al., 2007). Indeed, the mPFC is actively recruited and prominently contributes to the control of dominance hierarchy (Wang et al., 2011), spatial navigation (Benchenane et al., 2010), and social behavior (Lee et al., 2016). In these cases, both activity and connectivity of the mPFC have been associated with ongoing behavioral needs. However, little is known about the dynamics of the mPFC network during executive function in social contexts. Here, we tested the hypothesis that the connectivity of the mPFC correlates with specific features of social behavior and dominance hierarchy, particularly in dominant mice. We developed a simple social task and performed cortical recordings in acutely anesthetized and chronically implanted mice. Our results show that dominance hierarchy modulates social interactions during collective displacement and is reflected in the activity and connectivity patterns of the mPFC and hippocampus.

## Results

### Social interactions affect displacement patterns during goal-directed spatial behavior

**Figure 1:**
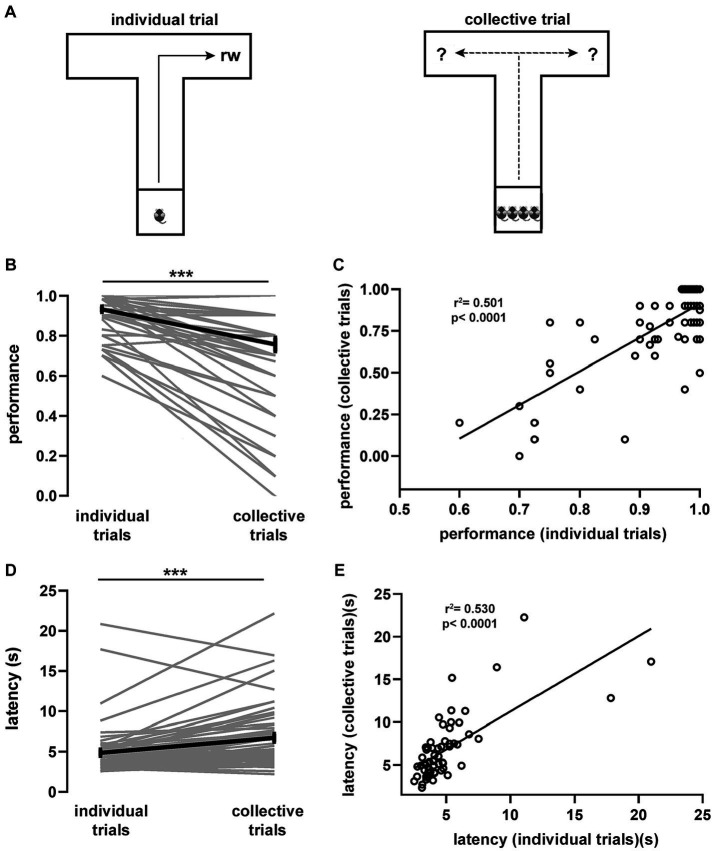
Spatial task and associated behavioral performance. **(A)** T-maze spatial task in two variants. Littermate mice (*n* = 60) were individually trained (10 trials per day) to navigate the maze foraging for food located at the end of an arm until reaching the learning criterion. Thereafter, four individual trials (reward in fixed location) were alternated with one collective trial (reward in random location). From every litter, two randomly chosen mice were consistently trained to look for food in one arm and the remaining two mice in the opposite arm. Average task performance **(B)** and latency **(D)** for individual mice during collective and individual trials sampled during the testing phase. **(C)** Spearman correlation between the average performance of individual trials against the average performance of collective trials. *R*^2^ = 0.501, *p* < 0.0001. **(E)** Spearman correlation between the average latency of individual trials against the average latency of collective trials. *R*^2^ = 0.530, *p* < 0.0001. Wilcoxon signed rank test (***, *p* < 10e-6). Gray lines, individual mice; black lines, population averages ± SEM; circles, individual mice average.

We first characterized the influence of the social environment on individual displacement patterns during collective behavior. For this, we trained groups of 4 male littermates in a spatial task, based on a modified version of the T-maze (Olton, 1979), which accommodated all mice simultaneously (Figure 1A). As a result, our T-maze was larger than standard versions (Supplementary Figure S1). We structured the task in 2 sequentially ordered phases consisting of a training phase (10 trials per day for each mouse) followed by a testing phase. During training, 2 littermates were pseudo-randomly assigned to individually look for reward exclusively in the left arm, while the right arm was baited for the 2 remaining littermates. There was large variance in performance (defined as the proportion of correct choices during individual trials) between individuals during the training phase (Supplementary Figure S2), but overall, mice improved their performance linearly over time (Supplementary Figure S3). In addition, they progressively decreased their latency, defined as the time interval taken to reach the rewarded pocket in the baited arm (Supplementary Figure S3). Throughout sessions, performance and latency co-varied linearly (Supplementary Figure S3) suggesting their interdependence. Littermates were sequentially trained on the same session, and since learning rates varied among mice, we defined a learning criterion based on litter performance (3 of 4 mice with 0.75 performance during 2 consecutive days).

Once the learning criterion was reached, mice started the testing phase, which consisted of 4 consecutive individual trials that were followed by a collective trial, in which all 4 littermates were tested simultaneously (Figure 1A). To prevent learning of the reward location during collective trials, arms were randomly baited (in every collective trial). We found that, during the testing phase, both performance and latency of individual trials reached a plateau and were stable over the remaining testing period, suggesting that the task had been acquired and consolidated (Supplementary Figure S3). Notably, comparison of performance during individual (0.93 ± 0.01) and collective (0.75 ± 0.03) trials showed a significant drop (*p* = 2.72e-08, Figure 1B), suggesting an important crowding effect in the movement patterns of individual mice during collective behavior. The performance drop in collective trials was largely determined by performance in the previous learning phase expressed in individual trials, as they were strongly correlated (*p* = 1.5e-10, Figure 1C). Conversely, task latency during the testing phase increased when comparing individual (4.99 ± 0.40 s) and collective (6.86 ± 0.49 s) trials (*p* = 4.86e-07, Figure 1D). Increased latency was proportional to the previous individual performance since mice exhibiting short latency during individual trials increased less their latency during collective trials (*p* = 4.4e-11, Figure 1E). Hence, the presence of other mice in the maze produced a shift in displacement patterns, which was reflected in performance decay and a proportional latency increase during goal-directed, collective spatial movement.

Movement decisions in animal groups often depend on contingent social interactions among individual subjects (Conradt and Roper, 2003; Couzin and Krause, 2003). During collective movement, animals tend to be attracted to conspecifics to avoid being isolated and to align themselves with neighbors (Partridge et al., 1980; Partridge, 1982). Thus, we reasoned that, during collective movement, mice might modify their previously learned trajectory depending on the distribution of animals in the maze arms. To test this idea, we calculated for every mouse the relative average density of animals located in the selected arm and projected it against its average performance during collective trials (*p* = 0.00016, Supplementary Figure S4). We found that, during collective trials, performance was inversely proportional to the relative density of animals in the selected arm. Conversely, there was no relation between the proportion of animals located in the opposite arm and performance during collective trials (*p* = 0.293, Supplementary Figure S4). This implies that the density of animals in the arm that a given mouse chose to move into was correlated with its task performance. To further explore this observation, we used a mixed logistic model to assess the influence of the spatial distribution of animals on task performance during collective trials (Supplementary Tables S1, S2). We confirmed that animal density in the arms exerted significant influence in shifting the movement strategy during collective trials, with particular relevance to the proportion of mice located in the selected arm (*p* = 6.41e-11). Thus, contingent social interactions were able to interfere with spatial displacement during collective trials.

### Dominance hierarchy defines the influence of the social condition during spatial movement

**Figure 2:**
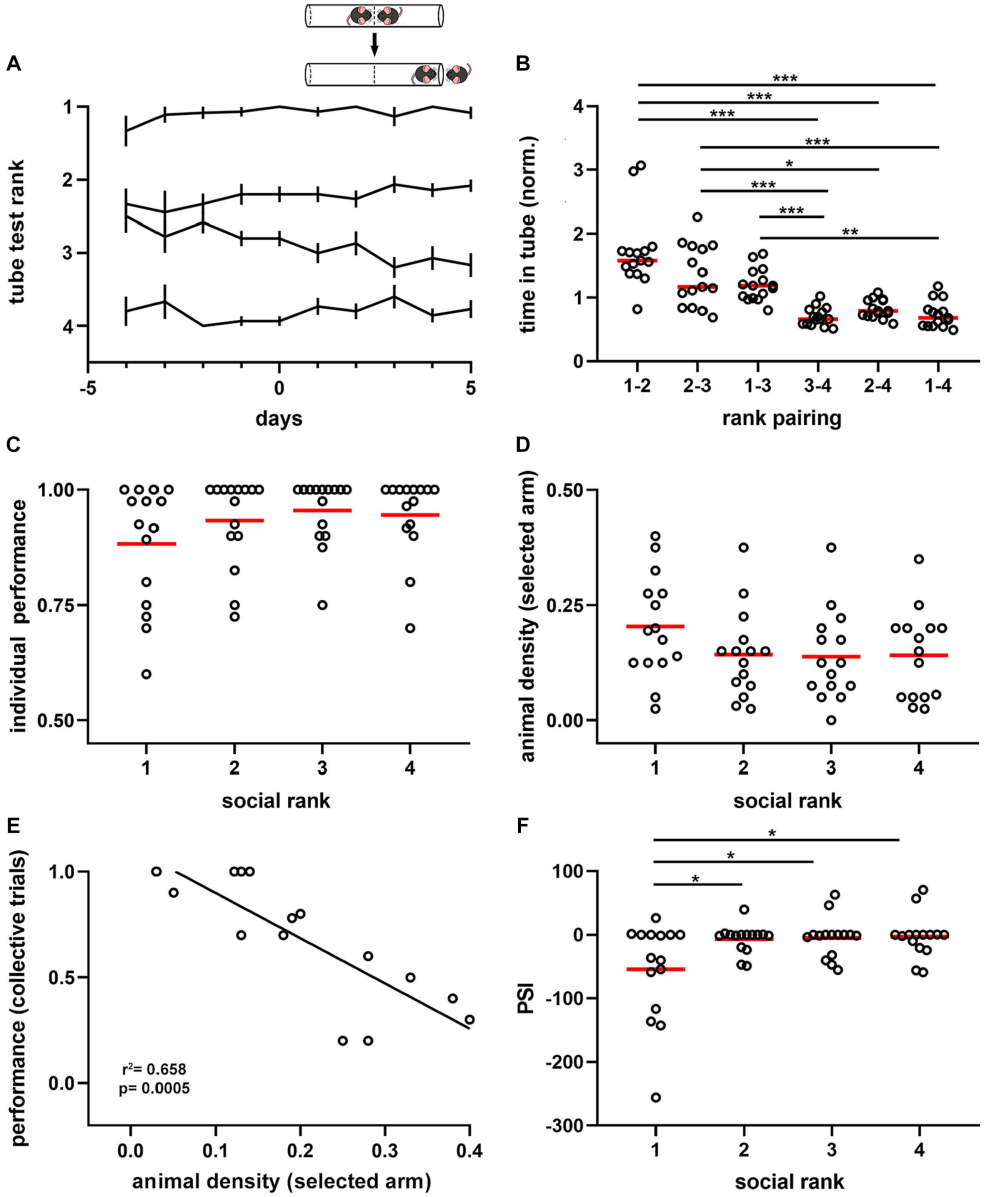
Dominance hierarchy and sensitivity to the crowding effect during spatial displacement. **(A)** Summary plot for all experimental cages (*n* = 15). Lines show average rank position based on the proportion of victories in the tube test (inset) correlative to the spatial test (days −4 to 0, training phase; days 1 to 5, testing phase). Ranking 1, dominant; ranking 2, first active subordinate; ranking 3, second active subordinate; ranking 4, submissive. Note ranking stability over time, particularly for dominant mice. Inset, schematic of the tube test used to identify the mice ranking system. **(B)** Normalized time spent in the tube for the six pairing conditions. One-way ANOVA, *p* = 2.31e-13. **(C)** Average performance on individual trials during the testing phase by social ranking. Kruskal-Wallis test, *p* = 0.251. **(D)** Animal density in the arm selected by animals during the spatial task according to social ranking. One-way ANOVA, *p* = 0.221. **(E)** Pearson correlation between the average performance of dominant mice in collective trials against the animal density in the selected arm. *R*^2^ = 0.658, *p* = 0.0005. **(F)** Peer sensitivity index (PSI) by social ranking. One-way ANOVA, *p* = 0.0084. Bonferroni test *post hoc* (*, *p* < 0.05; **, *p* < 0.01; ***, *p* < 0.001). Black lines, population averages ± SEM; circles, individual mice average; *red lines, population averages.*

We then explored the role of dominance hierarchy (Lindzey et al., 1961) on social interactions during spatial displacement. We assessed hierarchical relations of mice with the tube test (Wang et al., 2011) in parallel to the spatial displacement task described above. This test measures the dominance tendency by placing pairs of mice in a narrow tube, facing each other, and one mouse forces the other out backward to obtain victory (Figure 2A). We established social ranking based on the success rate of mice in pair-wise tests, using a round-robin design (Figure 2A), and found that the interaction time in the tube was shorter as the ranking difference between animals increased (*p* = 2.31e-13, *F* = 20.58, df = 5, Figure 2B). Dominance hierarchy was stable over time, particularly for the dominant mouse, whose position was rarely challenged throughout the experimental protocol (Supplementary Figure S5). Interestingly, the dominant mouse was not the largest animal in the group, as body masses were similar between rankings during both *ad libitum* food access (*p* = 0.8216, Supplementary Figure S6) and food restriction periods in the spatial test (*p* = 0.6554, Supplementary Figure S6). During the training phase, we observed that social ranking was not relevant for task acquisition as performance and latency were comparable between animals regardless of their dominance hierarchy (Supplementary Figure S3). Similarly, the time required to reach the learning criterion was not different between social rankings (*p* = 0.9609, Supplementary Figure S7). During the testing phase, the performance drop and latency increase in collective trials was not modulated by dominance hierarchy, as it was not different between social groups (performance, *p* = 0.6552; latency, *p* = 0.5662; Supplementary Figure S7).

Next, we assessed the influence of dominance hierarchy on task performance and found no significant difference between collective (*p* = 0.474) or individual (*p* = 0.2508, Figure 2C) trials across social ranks in the spatial task. Similarly, comparing dominant animals with all subordinate mice, showed no significant differences in task performance (Supplementary Figure S4). We then evaluated the effect of social contingent interactions arising during the spatial task, as this is another factor modulating performance. We compared animal density in the selected arm and found no significant difference across social ranks during collective trials (*p* = 0.2211, Figure 2D). Similarly, no difference was detected in the opposite arm (*p* = 0.4499, Supplementary Figure S4). Latency to reach the T-maze junction also showed no significant difference between social groups (*p* = 0.2371), suggesting that movement speeds were roughly similar between social rankings. Thus, both individual performance and animal density in the maze were not different between social groups; yet this was not informative about their interaction. To approach the relationships between animals, we assessed for every social ranking the influence of littermate distribution on task performance during collective trials. We found that task performance of dominant animals was uniquely influenced by the social group. Indeed, there was a negative correlation between the average density of animals located in the selected arm and the average performance during collective trials for dominant mice (*p* = 0.0005, Figure 2E), which was absent in the subordinate groups (Supplementary Figure S4). To obtain an estimate of the social influence on individual animals, we computed for every animal the regression coefficient of the spatial distribution of mice on the maze against task performance in the collective test and called it the ‘peer susceptibility index’ (PSI, median = 0, IQR = 35.13). Since PSI was proportional to the social influence on individual behavior, the larger its value, the stronger the crowding effect on task performance. Thus, negative values reflect a detrimental crowding effect, whereas positive values indicate a beneficial effect on task performance. Importantly, PSI was significantly different between dominant mice and subordinate groups (*p* = 0.0084, *F* = 20.58, df = 3, Figure 2F), thus suggesting that dominant mice were more likely to shift their decision based on the crowding effect. Moreover, differences in PSI did not result from different overall distributions of littermates in the maze during collective trials according to social ranking (*p* = 0.1783, Supplementary Figure S4). Altogether, these results suggest that mice exhibit differential susceptibility to contingent social interactions, outlined by dominance hierarchy.

### Activity and connectivity in the hippocampal-prefrontal circuit correlate with dominance hierarchy

Previous studies have established the neural basis of dominance hierarchy in the synaptic connectivity of the mPFC (Wang et al., 2011, 2014), hence we studied the relation between intrinsic cortical dynamics and social ranking system. We recorded spontaneous rhythmic cortical activity in animals with stereotaxically-implanted electrodes in mPFC and hippocampus (Supplementary Figure S8), two brain regions that are required for spatial navigation and social behavior (Negrón-Oyarzo et al., 2018). To focus on intrinsic activity and connectivity patterns during spontaneous cortical dynamics and minimize behavioral confounds resulting from different social ranking, we first performed experiments under deep anesthesia. To compare relatively similar conditions, we assessed the depth of anesthesia, as revealed by the power of the delta frequency band (0.5–4 Hz) of the mPFC and found no differences between social ranks (*p* = 0.081, Supplementary Figure S9). This result suggested that the global brain state was roughly similar across social groups.

**Figure 3:**
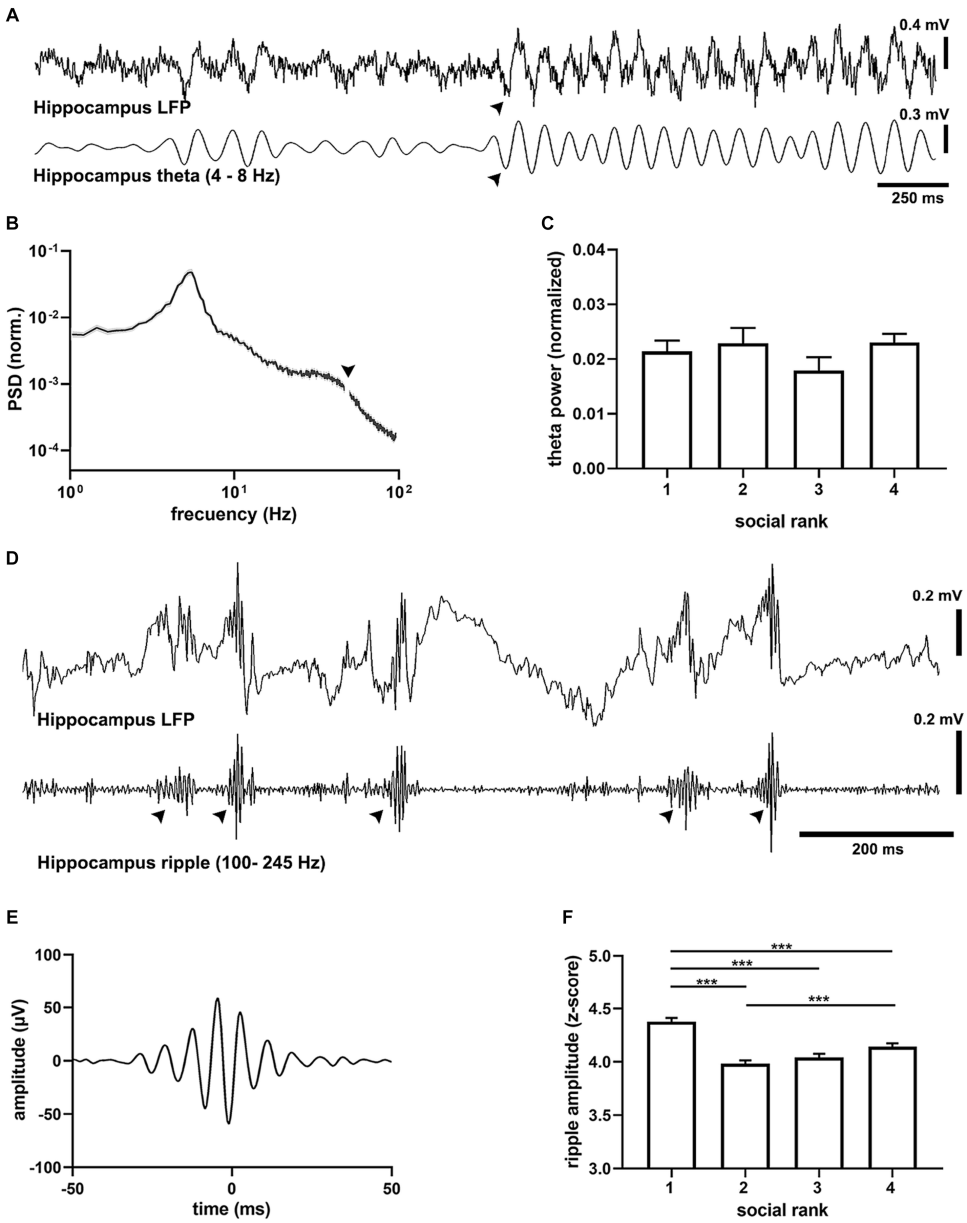
Oscillatory activity in the dorsal hippocampus. **(A)** Example recording of CA1 activity showing theta oscillations (filtered 4–8 Hz) recorded in a urethane-anesthetized mouse (CM24reg05). Note prominent transitions into theta activity (arrowhead). **(B)** Average power spectral density (PSD) from all recordings (*n* = 22 mice). Arrowhead points to the removed mains 50 Hz artifact. **(C)** Peak hippocampal theta amplitude by social ranking. One-way ANOVA, *p* = 0.3811. **(D)** Example recording of hippocampal (LFP) showing hippocampal sharp wave ripples (SWRs, filtered 100–250 Hz) in a urethane-anesthetized mouse (CM75reg06). **(E)** Grand average ripple episode (*n* = 18,571 events, 22 animals). **(F)** Peak SWRs amplitude by social ranking. One-way ANOVA, *p* = 8.55e-18.

Analysis of the hippocampal oscillations showed that all animals exhibited epochs of theta-band oscillations (4–8 Hz) that alternated with spontaneous, prominent sharp wave-ripples (SWRs, 100–250 Hz) characteristic of hippocampal exploratory (Buzsáki, 2015), quiescent (Buzsáki and Moser, 2013) states and can be mimicked by urethane anesthesia, respectively (Figure 3). We first assessed activated cortical states that were characterized by prominent theta oscillations (Buzsáki, 2002). The spectral distribution of field potentials evidenced strong hippocampal theta oscillations (Figure 3B) that were consistently similar between social groups (Figure 3C), with comparable frequency and duration (Supplementary Figure S10). Since oscillatory synchrony is a neural mechanism for functional coupling of distributed neural circuits (Jones and Wilson, 2005), we assessed the spontaneous spectral coherence in the field potential activity of the hippocampal-cortical circuit. We identified elevated intercortical coherence in theta oscillations under anesthesia in all animals, with no difference between social rankings (Supplementary Figure S10). We then probed the quiescent states of the brain network (Figure 3D), when SWRs dominate hippocampal activity (Ylinen et al., 1995), and found prominent SWR events, which occurred as short-lived, fast waxing and waning oscillations (Figure 3E). SWR amplitude was dependent on social ranking as dominant mice exhibited the largest SWRs when compared to the subordinate groups (*p* = 8.55e-18, *F* = 27.59, df = 3, Figure 3F). Differences in SWRs’ amplitude was not the result of variability in the LFP signal as the standard deviation of the ripples band was not different between social rankings (Supplementary Figure S11). The duration of SWRs was not different between social ranks (*p* = 0.0658), yet their frequency was slower in the submissive group (*p* = 5.45e-38, *F* = 59.06, df = 3, Supplementary Figure S11).

**Figure 4:**
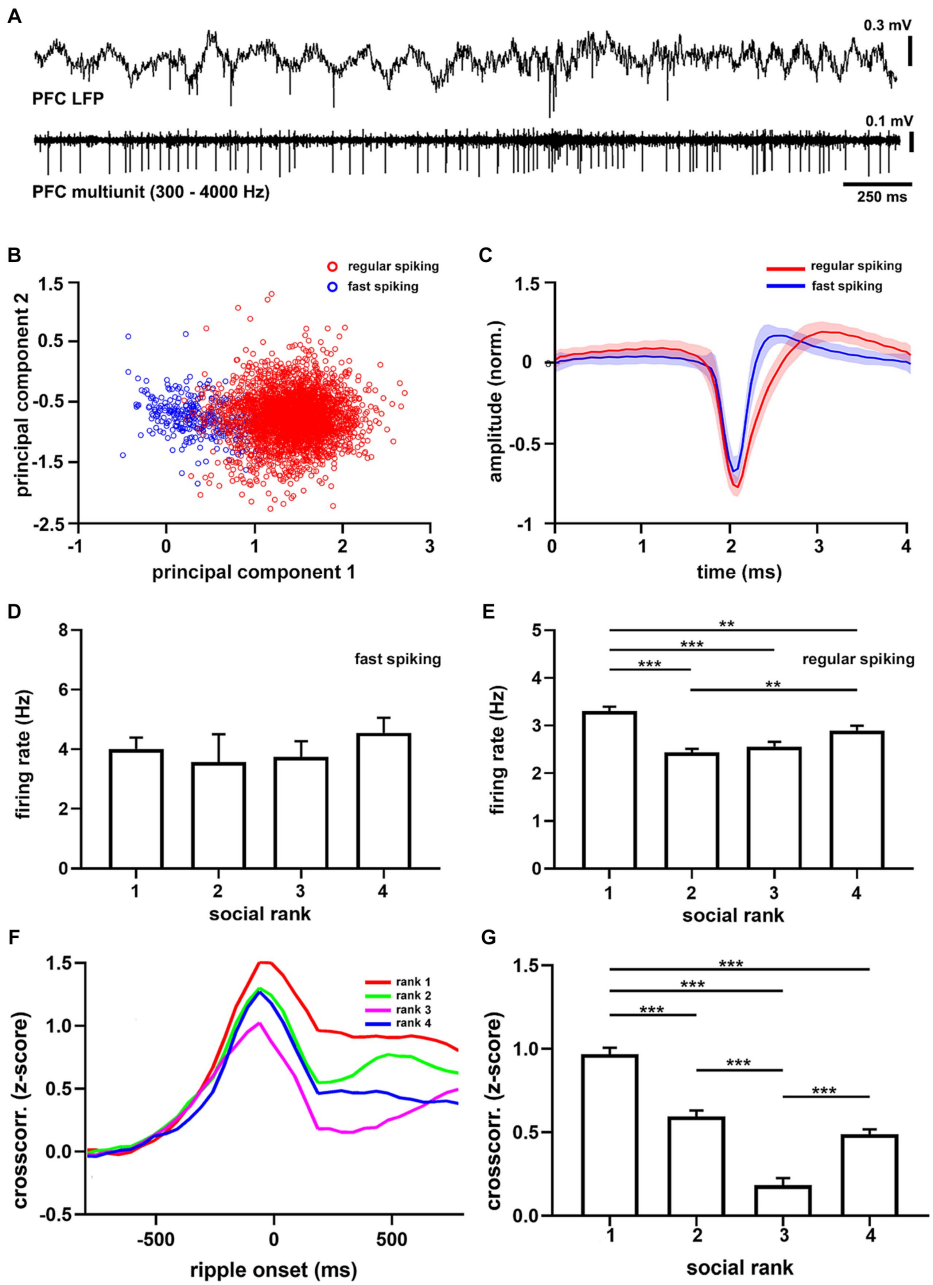
Medial prefrontal cortex units recorded under anesthesia. **(A)** Example recording of medial prefrontal cortex activity (PFC LFP) and multiunit activity (PFC multiunit, filtered 300–4,000 Hz) recorded in a urethane-anesthetized mouse (CM24reg05). **(B)** Principal components from all recorded units (*n* = 3,702 units). Red shows largest cluster, regular spiking cells; blue shows smallest cluster, fast spiking cells. **(C)** Grand average of regular spiking (red, *n* = 3,382) and fast spiking (blue, *n* = 320) units. Firing rate sorted by social ranking for fast spiking (**D**, *p* = 0.6243) and regular spiking (**E**, *p* = 6.07e-12) units. One-way ANOVA. Note that regular spiking cells in dominant animals discharge more than subordinate groups. **(F)** Average crosscorrelograms between the onset of sharp wave ripples and prefrontal units (*n* = 22 animals) sorted by social ranking (*n* = 3,702 units). **(G)** Crosscorrelogram amplitude by social ranking. One-way ANOVA, *p* = 7.71e-47.

Slice recordings in the mPFC have shown that dominant animals exhibit larger synaptic strength in their excitatory synapses than submissive mice (Wang et al., 2011). We thus tested whether this *in vitro* relation translated to *in vivo* spiking patterns (Figure 4A; Supplementary Figure S12) and found that the overall mPFC firing rate in dominant mice was larger than the subordinate groups (*p* = 5.56e-11, Supplementary Table S3). Interestingly, when clustering units into different types (Figures 4B,C), it became apparent that the difference was specific to regular spiking cells (*p* = 6.07e-12, *F* = 18.57, df = 3, Figure 4E), which are putative pyramidal neurons, as it was not detected in fast spiking units (*p* = 0.6243, Figure 4D), which are putative interneurons. Hence, dominant mice exhibited larger levels of intrinsic spiking activity in the mPFC under anesthesia and did not correlate with social ranking in general.

Next, we studied ripples as they powerfully synchronize neuronal spike-timing across the neocortex (Logothetis et al., 2012; Remondes and Wilson, 2015), including the mPFC (Siapas and Wilson, 1998). Therefore, we computed cross-correlation functions between hippocampal SWRs and mPFC spikes to quantify their degree of synchronization. We found that following SWR episodes, cortical activation was evident as a prolonged after-discharge of mPFC neurons (Supplementary Figure S13). Notably, ripple after-discharges were stronger in dominant mice when compared to subordinate groups (Figure 4F), suggesting enhanced functional connectivity following SWR episodes (*p* = 7.7e-47, *F* = 74.59, df = 3, Figure 4G). These observations were robust, confirmed by shuffling comparisons, and apparent when assessing the entire neuronal population (*p* = 2.81e-07, *F* = 11.37, df = 3,). Moreover, the differences in after-discharges were not the result of different temporal distributions of SWRs between social ranking, as inter-ripple intervals were similar across social groups (*p* = 0.6193, Supplementary Figure S11). Altogether, our results show that dominance hierarchy is associated with distinct cortical activity and connectivity patterns, with the dominant mice exhibiting larger mPFC firing rates, larger hippocampal SWR episodes, and stronger coupling between mPFC neurons and hippocampal SWR episodes.

### Spiking activity in prefrontal cortex signals the social condition according to dominance hierarchy

**Figure 5:**
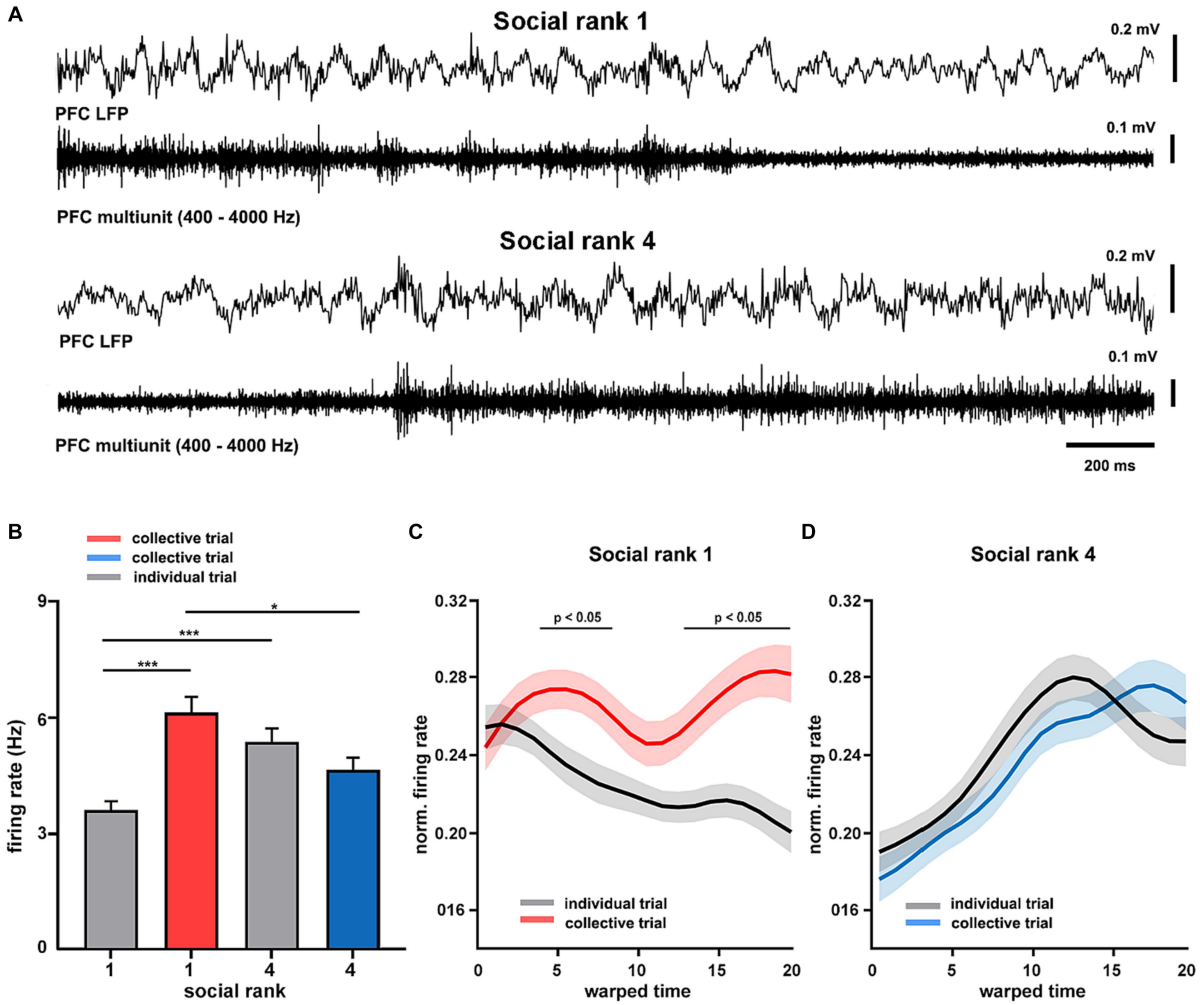
Cortical dynamics of dominance hierarchy during spatial displacement. **(A)** Example recordings of prefrontal cortical (LFP PFC) activity showing theta oscillations (filtered 4–8 Hz) and neuronal spiking (units PFC, filtered 300–4,000 Hz) from two chronically-implanted dominant (rank 1, mouse H8372) and submissive (rank 4, mouse H8373) mice during spatial displacement in the T-maze. **(B)** Firing rates of mPFC single units (*n* = 189 from 8 animals) according to social condition and social ranking. Two-way ANOVA, *p* = 10e-6 (condition*ranking) and *p* = 0.0065 (condition). Normalized firing rates for dominant (**C**, *n* = 4 mice) and submissive (**D**, *n* = 4 mice) animals during T-maze task performance. Warped time represents entire T-maze. Bin 0, start box; bin 20, arm end. Asterisks depict significant differences between curves, Wilcoxon rank-sum (*, *p* < 0.05). Bonferroni test *post hoc* (*, *p* < 0.05; **, *p* < 0.01; ***, *p* < 0.001). Bars, average ± SEM; colored lines, average population; shading areas, ± SEM.

Having found distinct differences in intrinsic cortical dynamics between dominant and subordinate animals, we then sought to study the spike timing of the mPFC during collective behavior. To that end, we implanted tetrodes in the mPFC of dominant and submissive mice and recorded their single-unit activity during goal-directed behavior (Supplementary Figure S14). Here, we further simplified the T-maze protocol, recording only during training sessions and using pairs of animals for collective trials (see Materials and Methods). During movement in the maze, the mPFC of freely-moving animals exhibited prominent theta oscillations, similar to those recorded under anesthesia, modulating the spike timing of cortical neurons in both dominant and submissive mice (Figure 5A). We focused on the firing patterns of individual mPFC units and compared them across social ranking and social condition. We detected a significant interaction in which the social condition affected dominant animals by increasing their cortical spiking activity (*p* = 10e-6, *F* = 24.15, df = 1, Figure 5B). That is, the firing rate of mPFC neurons increased in dominant mice during spatial displacement in the presence of their littermates. Nonetheless, there were inherent differences in mPFC unit firing rates between dominant and subordinate mice even during individual trials. Given that trial durations were not significantly different between according to social condition (*p* = 0.2217, Supplementary Figure S15), this result is unlikely to be explained by different movement speeds. Similarly, task performance was similar between collective and individual tasks. Thus, this is not a plausible modulator of spiking activity (*p* = 0.2597, Supplementary Figure S15). In addition, firing rates of individual neurons were consistent across trials and exhibited little variation between recording sessions, but maintained a noticeable difference between social conditions (Supplementary Figure S15). Next, to compare the temporal evolution of spiking activity during spatial displacement we normalized both firing rates and trial durations. In the individual trials, the neuronal activity profile of dominant mice was distinctively different according to the social condition as neuronal spiking progressively decreased, whereas it irregularly increased in collective trials (*p* < 0.05, Figure 5C). Conversely, the mPFC neurons of submissive animals discharged with similar dynamics regardless of the social condition (*p* > 0.05, Figure 5D). Overall, these results confirm that neuronal activity in the mPFC of dominant animals is sensitive to the social condition.

## Discussion

Our results show that goal-directed spatial behavior acquired individually can be disrupted by contingent social interactions arising during collective trials. Performance in the social condition was critically dependent on both the previous individual learning process, during the training phase, and contingent social interactions occurring during spatial movement, in the testing phase. Ultimately, the influence of contingent social interactions on individual behavior depended on dominance hierarchy and correlated with the intrinsic connectivity of the hippocampal-prefrontal circuit. Moreover, neural spiking in the mPFC partly correlated with social performance during both spatial displacement and dominance behavior. Hence, intrinsic cortical activity and connectivity patterns seemingly differentiate dominance hierarchy and social behavior.

Given that trained animals acquired complete information about the spatial task during the training phase, it might be expected that mice would maintain their stereotyped, efficient movement strategy during the testing phase of the spatial task. Instead, animals flexibly switched strategies and privileged multimodal evidence arising from contingent social interactions, which resulted in the significant loss of performance and increased latency during collective movement. The shift in displacement strategy was not directly related to dominance hierarchy, but to contingent social interactions arising during collective movement. Indeed, task performance of dominant mice in collective trials was significantly correlated with the distribution of animals in the maze, regardless of the previously learned reward location during individual trials. Recent studies have shown that as experience increases, mice shift their sensory-based strategy to more efficient, stereotyped foraging based on spatial memory that varies little in response to sensory cues (Gire et al., 2016). Conversely, our results show that when experiencing the crowding effect, it can substantially modify the individual behavior of dominant animals. Perceptual evidence arising in the social condition is possibly more elaborate than common individual experience as it recruits all sensory modalities and specifically activates circuits for the recognition of and interaction with conspecifics (Insel and Fernald, 2004). For example, there is a strongly correlated group structure among mice, as more information about group behavior is contained in the joint position of mice than what can be extracted from summing all the information provided by the interactions between pairs of mice (de Chaumont et al., 2012; Shemesh et al., 2013). This irreducible high-order structure in social behavior further supports the study of collective behavior in social groups instead of focusing on individuals.

Ranking systems emerge in social groups to regulate competition over limited resources. Rodents establish dominance hierarchy based on a history of recurrent social interactions in which one subject acts as dominant over the subordinate individuals. Dominance hierarchy can be assessed and quantified by several tests, including the agonistic behavior assay, the barber test, and the ultrasonic test, among others. Here, we used the tube test that is consistent with all those paradigms and has been validated based on transitivity, consistency, and stability (Wang et al., 2011). In agreement with previous reports, we found that dominance hierarchy was stable over time (Wang et al., 2011). Interestingly, dominance hierarchy had no direct relation with task performance during collective behavior; yet, it distinctly modulated the susceptibility of individuals to contingent social interactions arising during spatial movement. Previous studies have shown that effective leadership and social decision-making during collective movement do not require intrinsic differences between individuals, such as dominance hierarchy or body size (Couzin et al., 2005). This striking behavioral pattern suggests that, in the social condition, dominant mice shift their interest from performing the goal-directed task to monitoring the collective behavior of the social group. This pattern is completely at odds with the acquisition of the spatial task, in which dominant and subordinate groups showed comparable performance and latency. Moreover, intrinsic cortical dynamics revealed significant differences according to social ranking, as dominant mice exhibited distinct intrinsic cortical activity and connectivity patterns that segregated them from the subordinate groups, with larger mPFC firing rates, larger hippocampal SWR episodes, and stronger coupling between mPFC neurons and hippocampal SWR episodes.

An important limitation of our study is that we could not track individual trajectories of all littermates during collective trials. Hence, we do not have detailed information about their instantaneous locomotion speed or movement patterns. Cortical oscillations are strongly state-dependent, and lacking such data may be relevant since human and animal experiments support a role for the hippocampus in imagination, planification, and memory retrieval (Ylinen et al., 1995; Joo and Frank, 2018). Importantly, SWRs dominate hippocampal activity during quiescent states (Ylinen et al., 1995); for example, when animals exploring the environment make a pause or stop, and those moments may be highly relevant for temporal prospection or planning (Jadhav et al., 2012). Indeed, hippocampal SWRs precede successful memory retrieval in awake humans (Vaz et al., 2019) and have been proposed to support decision-making and imagination (Joo and Frank, 2018). Importantly, hippocampal-prefrontal coordination during SWRs has also been proposed as a neural substrate for decision-making (Yu and Frank, 2015). Indeed, hippocampal spiking during SWRs can represent past or potential future experience (Joo and Frank, 2018), and ripple disruptions affect memory performance (Girardeau et al., 2009; Jadhav et al., 2012). Hence, SWRs support both memory consolidation and memory retrieval, which could be at the service of associated cognitive processes such as decision-making. We have previously shown that adverse environmental conditions, such as stress, impair intrinsic hippocampal-cortical connectivity following SWRs. Importantly, such disruptions are accompanied by alterations in long-term memory (Negrón-Oyarzo et al., 2015). Thus, our current results suggest that the strength of intrinsic hippocampal-cortical connectivity is a potential candidate for the modulation of goal-directed behavior in social groups. Moreover, we found enhanced activity and connectivity in the hippocampal-prefrontal circuit, yet it is not clear from our experiments whether this is specific or just a generalized increase of activity in the entire brain of dominant animals. Our results showing comparable cortical slow wave oscillations between social ranks suggest similar overall activity patterns in the cortical mantle, yet further experiments will have to assess this issue in detail.

Social relationships can shape individual behavior and affect decision-making (Torquet et al., 2018). Although dominance hierarchy emerges from recurrent social interactions, it causally results from the synaptic efficacy of excitatory transmission in the mPFC (Wang et al., 2011). The mPFC is essential for decision-making, executive behavior, and social interactions (Euston et al., 2012). We report here that the internally generated, self-organized patterns of cortical activity, unrelated directly to behavior or relevant perceptual processing, may define the framework of behavioral performance. Naturally, this observation cannot fully account for the shifting in decision-making in the social condition, as other cortical regions also contribute to goal-directed spatial navigation. For example, the orbitofrontal cortex is relevant in shifting decisions (Gremel and Costa, 2013). Indeed, previous studies have established that orbitofrontal circuits encode the shift between goal-directed and habitual actions (Gremel and Costa, 2013), thus allowing flexible and efficient decision-making. This is also consistent with recent findings showing that the orbitofrontal cortex integrates prior (i.e., memory) with current (i.e., sensory) signals to guide adaptive behavior (Nogueira et al., 2017). The mPFC exhibits robust anatomical connectivity with the orbitofrontal cortex (Euston et al., 2012), and these reverberant connections are certainly important in shifting decision-making strategies. Thus, to further understand decision-making in the social condition, future studies will have to assess not only ongoing activity of the prefrontal cortex during decision-making and spatial navigation, but also the contribution of intrinsic network dynamics in other functionally connected cortical regions. Overall, our results suggest that hippocampal-cortical activity and connectivity patterns are important factors to define dominance hierarchy and consequent social behavior. These results suggest that the interplay between contingent social interactions and dominance hierarchy can regulate behavioral performance, supported by the coordinated activity of the hippocampal-prefrontal circuit.

## Methods

### Ethics statement

All methods were performed in accordance with the relevant guidelines and regulations. All procedures involving experimental animals were reviewed and approved by university (Comite Etico Cientifico para el cuidado de animales y ambiente, CEC-CAA) and national (Comision Nacional de Investigacion Cientifica y Tecnologica, CONICYT) bioethics committees. Efforts were performed to minimize the number of animals used and their suffering. All procedures involving experimental animals were performed in accordance with ARRIVE guidelines, reviewed and approved by the Institutional Animal Ethics Committee of the Pontificia Universidad Catolica de Chile (protocol code CEBA-13-040).

### Animals

Groups of four male sibling mice (C57BL/6 J strain, 20–30 g, Supplementary Table S4) were reared together after weanling. All tests were conducted between 10.00 a.m. and 4.00 p.m. Animals were housed under controlled temperature (22 ± 1°C) and humidity (50%) conditions with food and water *ad libitum*. A 12 h:12 h light–dark cycle was maintained throughout experiments, lights being on from 8.00 a.m. to 8.00 p.m.

### Tube test

We measured hierarchical relations within social groups with the tube test (Lindzey et al., 1961). Each mouse was placed at the ends of a narrow tube facing inward and one mouse forces the other to back out of the tube with score 1 for the winner and 0 for the loser per session (Wang et al., 2011). All mice were tested pairwise for dominance for ten consecutive days using a round-robin design, and the social rank was assessed based on winning against the other cage mates (Supplementary Video S1).

### T-maze test

For habituation, animals (60 in total, coming 15 cages of 4 littermates each) were individually placed in the maze and allowed to explore freely for 3–5 min for 2 consecutive days. Crumbs of sweet cereal were randomly distributed throughout the maze to stimulate exploration. Next, we repeated the procedure, but animals from the same cage (4 littermates) were collectively placed in the maze and allowed to explore freely for 3–5 min for two consecutive days. Animals were then food-restricted (with 1.5 gr of pellet per animal per day) to enhance exploration, and learning to identify and move towards the arm baited with food. This treatment produced a general weight loss of about 20% in most animals.

During the individual phase, we trained animals individually to look for food, a small piece of commercial sweetened cereal (50–100 mg each) placed in a fixed location at the end of one of the maze arms. Each animal performed 10 trials per day, with a maximum duration of 90 s per trial. During the first trials, if the mouse did not explore, it was gently pushed towards the baited arm. Animals were manually transferred from the baited arm to the start box in every trial. They were not directly grabbed, but allowed to enter a small cardboard cylinder (usually placed in the homecage) used to move them between homecage and maze. At the end of every trial, the mouse was placed back in the homecage with its siblings and the maze was quickly wiped with 10% EtOH to remove odour cues. In this way, the inter-trial interval for every mouse was around 10 min. At the end of all trials for all littermates, mice were put back in the homecage and cereal crumbs were scattered throughout, so all mice had access to cereal. For every trial we computed the latency or the time interval that every animal took to get from the start box to reach the food reward in the baited arm. In addition, we calculated the performance as the proportion of correctly performed trials based on the first decision to turn to the baited arm. Training finalized when animals reached the learning criterion, meaning that at least three out of four littermates in the box performed correctly six out of eight trials (75%) on two consecutive days. Importantly, using a more stringent criterion, such as 80% correct trials, did not significantly affect the results of the study (Supplementary Figure S16). After reaching the learning criterion, animals started the collective phase.

In the collective phase, every animal performed four consecutive individual trials, followed by one collective trial, with all four littermates placed in the start box. This was repeated twice, so as to complete 10 trials in total (8 individual and 2 collective trials per day), during 5 consecutive days. During collective trials, reward was randomly assigned according to four possible options: right arm (1 piece of cereal), left arm (1 piece of cereal), both arms (1 piece of cereal in each arm), or none (0 piece of cereal). Once the animals had completed the task (10 trials in total), they were transferred back to the homecage and received scattered reward. In the case of implanted animals, they were individually fed in their cage.

We used transparent Plexiglas to manufacture the T-maze. The body weight of animals was monitored daily during the test sessions (Supplementary Figure S6). Reward location was randomized in the collective trials in order to prevent learning of food location. In this way, reward-seeking behavior would be guided only from memory acquired during individual trials. The animal density was computed across the entire maze (central arm, selected arm, and opposite arm). Thus, the sum of the relative densities in the lateral arms does not necessarily account for all mice, as they could also be located in the central arm. Videos of individual and collective tests were scored manually on a frame-by-frame basis (Supplementary Videos S2, S3). The experimenter was blind to the animal identity during scoring.

### Acute surgery

After finishing collective testing, animals were allowed to recover *ad libitum* weight and then were used for electrophysiological recordings. Procedures were similar to what we have previously described (Negrón-Oyarzo et al., 2015; Espinosa et al., 2019a,b). We recorded simultaneous neuronal activity in the prefrontal cortex and hippocampus. Animals were anesthetized with urethane (0.8 g/kg dissolved in saline, i.p.) and a mixture of ketamine/xylazine (40 mg/kg ketamine; 4 mg/kg xylazine dissolved in saline, i.p.). Anesthesia was maintained throughout the experiment with urethane administered every 20 min with a bomb when required. During the entire experiment, glucosamine solution (0.5–1 mL) was injected subcutaneously every 2 h to maintain the animal hydrated and body temperature was maintained at 36 ± 1°C using a homeothermic blanket (Harvard Apparatus, MA, United States) and monitored with a rectal probe connected to a temperature controller (Harvard Apparatus, MA, United States). Animals were firmly placed in a stereotaxic frame (Stoelting Co.).

### Acute recordings

Procedures were similar to what we have previously described (Negrón-Oyarzo et al., 2015). To simultaneously record neuronal activity of the prefrontal cortex (cingulate and prelimbic cortex) and the CA1 area of the dorsal hippocampus, small craniotomies (1 mm) were drilled on the skull (right hemisphere) over the recording sites. The stereotaxic coordinates, indicated by the stereotaxic atlas (Paxinos and Franklin, 2019), were (relative to bregma): prefrontal cortex, anteroposterior, +2 mm; mediolateral, +0.5 mm; and CA1 hippocampus, anteroposterior, −3 mm; mediolateral, +1.7 mm. The electrodes were slowly lowered via a motorized microdrive (Siskiyou, Grants Pass, OR, United States) to the recording positions. The electrodes were positioned at ∼1.0–2.0 mm dorsoventrally to record activity in the PFC and to record in the CA1 the electrodes were placed at ∼1.1 mm dorsoventrally using the firing of CA1 pyramidal cells and the appearance of SWR as the hallmark for functional localization of the hippocampus. Neuronal activity in the prefrontal cortex was recorded extracellularly with a 32 channel-two shank silicon probe (Poly A32, Neuronexus, mean site resistance ~1 MΩ) stained with DiI. Neuronal activity in the hippocampus was recorded with a 16 channel-silicon probe (A16, Neuronexus, mean site resistance ~1 MΩ) stained with DiI and inserted into the brain with a 30° angle through the midline. Electrical activity was acquired with a 32-channel Intan RHD 2132 amplifier board connected to an RHD2000 evaluation system (Intan Technologies). Single-unit activity and local field potential (LFP; sampling rate 20 kHz) were digitally filtered between 300 Hz −5 kHz and 0.3 Hz – 2 kHz, respectively. Once a spiking multiunit was detected, the simultaneous prefrontal cortex and hippocampal recording started, and lasted for 10 min.

### Chronic surgery

A survival surgery was conducted on adult mice (3–6 months of age), weighing 21–25 g. Animals were anesthetized with 1.5–2.5% isoflurane, delivered with O2 (2 L per min), and were placed in a stereotaxic frame. Using a surgical drill (Foredom Electric, Bethel, CT), two craniotomies were made, one on the occipital bone for a brass ground screw and another above the frontal bone for the tetrode array. The dura mater was removed for the following craniotomy. Mice were implanted with customized microdrives weighing ~1.5 g, including the dental cement. The array was targeted to the right PFC area, using the coordinates anteroposterior, +2 mm; mediolateral, +0.5 mm, and lowered to an initial depth of 1,500 μm below the brain surface. The exposed craniotomy was filled with sterile Vaseline. As the dental acrylic hardened, mice were injected with buprenorphine (0.05 mg/kg) subcutaneously prior to the removal of anesthesia. Mice were closely monitored for the first few hours post-surgery and were monitored daily thereafter.

### Chronic recordings

After the recovery period, the implanted animals (8 animals, 4 dominant and 4 submissive mice) were re-entrained individually to start the test phase. We recorded neural activity via a unitary gain headstage preamplifier (HS-18; Neuralynx Bozeman, MT), which was connected to an amplifier (Cheetah 32, Neuralynx) linked to the acquisition software (Cheetah 32, Neuralynx). Single units were recorded at a sampling rate of 30 kHz, band-pass filtered (600–6 kHz), and referenced to a nearby 50 μm local reference electrode in corpus callosum above dorsal CA1. Local field potentials were also acquired at a sampling rate of 3 kHz, band-pass filtered (0.1–6 kHz), and referenced to a ground screw above the cerebellum. This was accomplished by a video camera, mounted above the chamber, to the video input of the Cheetah software.

### Histology

Procedures were similar to what we have previously described (Negrón-Oyarzo et al., 2015). At the end of the electrophysiological recording mice were immediately perfused with 20 mL of saline solution followed by 50 mL of 4% paraformaldehyde in phosphate buffered saline (PBS, pH = 7.4). The brain was removed, incubated overnight in 4% paraformaldehyde in PBS buffer and then stored in PBS buffer containing 0.2% sodium azide. Coronal brain slices (60–80 μm) were prepared from paraformaldehyde-fixed brains with a vibratome (World Precision Instruments, Sarasota, United States) in ice-cold PBS buffer. For visualization, slices were washed three times in PBS buffer at room temperature and then placed on slides using a mounting medium (Dako) and then, were stained with Nissl-staining, Images were acquired with an epifluorescence microscope for DiI labelling and Nissl-staining (Nikon eclipse Ci).

### Spike sorting

Procedures were similar to what we have previously described (Negrón-Oyarzo et al., 2015; Espinosa et al., 2019a). Neuronal spikes were extracted from prefrontal cortex recordings using semiautomatic clustering KlustaKwik.^1^ This method was applied over the 32 channels of the silicon probe, grouped in eight pseudo-tetrodes of four nearby channels. Spike clusters were considered single units if their auto-correlograms had a 2 ms refractory period, and their cross-correlograms with all other clusters did not have sharp peaks within 2 ms of 0 lag. Details from all recorded units are presented in Supplementary Table S5.

### Brain-state and time-frequency analysis

We defined brain-states based on the hippocampal LFP (Negrón-Oyarzo et al., 2015; Espinosa et al., 2019a). Decomposition of LFP in PFC and hippocampus was performed with multi-taper Fourier analysis (Mitra and Pesaran, 1999) implemented in Chronux toolbox.^2^ LFP was downsampled to 500 Hz before decomposition. We recognized theta oscillations, non-theta epochs, and ripple episodes. Unless stated, the LFP from dorsal CA1 stratum pyramidal was considered as the time-frame reference for the spike-timing of recorded cells.

Theta oscillations were detected by calculating the continuous ratio between the envelopes of theta (4–8 Hz) and delta (2–3 Hz) frequency bands filtered from the hippocampus LFP and calculated by the Hilbert transform. A ratio of 1.4 SD or higher, during at least 2 s defined epochs of theta oscillations. Recording episodes outside theta oscillations were defined as non-theta epochs.

Sharp wave-ripples were recorded in dorsal CA1, as close as possible to stratum pyramidale (Supplementary Figure S17) and considered as the time-frame reference for the spike-timing of the recorded neurons and population activity (LFP) in prefrontal cortex. We used a recently described method for ripples detection (Logothetis et al., 2012) with some modifications. Briefly, the hippocampus LFP was first down-sampled to 1 kHz, then band-pass filtered (100–250 Hz) using a zero-phase shift non-causal finite impulse filter with 0.5 Hz roll-off. Next, the signal was rectified, and low-pass filtered at 20 Hz with a 4th order Butterworth filter. This procedure yields a smoothed envelope of the filtered signal, which was then z-score normalized using the mean and SD of the whole signal in the time domain. Epochs during which the normalized signal exceeds a 3.5 SD threshold were considered as ripple events. The first point before threshold that reached 1 SD was considered the onset and the first one after threshold to achieve 1 SD as the end of events. The difference between onset and end of events was used to estimate the ripple duration. We introduced a 50 ms-refractory window to prevent double detections. To precisely determine the mean frequency, amplitude, and duration of each event, we performed a spectral analysis using Morlet complex wavelets of seven cycles. The Matlab toolbox used is available online as LAN toolbox.^3^

### Cross-correlation analysis

Procedures were similar to what we have previously described (Negrón-Oyarzo et al., 2015; Espinosa et al., 2019a,b). Activity of spiking neurons and hippocampal ripples was cross-correlated by applying the “sliding-sweeps” algorithm (Abeles and Gerstein, 1988). A time window of ±1 s was defined with the 0-point assigned to the start time of a ripple. The timestamps of the cortical spikes within the time window were considered as a template and were represented by a vector of spikes relative to t = 0 s, with a time bin of 50 ms and normalized to the total number of spikes. Thus, the central bin of the vector contained the ratio between the number of PFC spikes elicited between ±25 ms and the total number of spikes within the template. Next, the window was shifted to successive ripples throughout the recording session, and an array of recurrences of templates was obtained. Both prefrontal cortex timestamps and start times of ripples where shuffled (1,000 samples) samples by randomized exchange of the original inter-event intervals and the cross-correlation procedure was performed on the pseudo-random sequence.

### Statistics

We performed inter-subject comparisons to establish if behavior and simultaneous cortico-hippocampal activity were different across social rank. We pooled neuronal data from all animals of a specific social rank in the same experimental group for all other statistical analysis. Data were tested for normality using the Kolmogorov–Smirnov test and then compared with the appropriate test with parametric analysis (one-way ANOVA followed by Bonferroni post-hoc test). Comparison between behavioral parameters and other non-normally distributed parameters were analysed with non-parametric tests (Wilcoxon signed rank; Kruskal-Wallis test followed by Dunn’s multiple comparisons post-hoc test). The statistical significance of the observed repetition of spike sequences was assessed by comparing, bin to bin, the original sequence with the shuffled sequence. An original correlation sequence that presented a statistical distribution different from 1,000 permutations was considered as statistically significant, with *p* < 0.01 probability, instead of a chance occurrence. Linear correlations between parameters were analysed by Spearman correlation test. To calculate the *p*-value we used the circ_corrcl.m in the CircStat toolbox of MATLAB (The Mathworks, Inc.) and STATISTICA 7.0 software (StatSoft, Inc).

## Data availability statement

The raw data supporting the conclusions of this article will be made available by the authors, without undue reservation.

## Ethics statement

The animal studies were approved by Comite Etico Cientifico para el Cuidado de Animales y Ambiente de la Pontificia Universidad Catolica de Chile. The studies were conducted in accordance with the local legislation and institutional requirements. Written informed consent was obtained from the owners for the participation of their animals in this study.

## Author contributions

AL-V, NE, CrM, JG, and JS performed experiments. AL-V, PB, PH, and PF designed experiments. AL-V, NE, PB, and PF analysed data. PF and PH wrote the manuscript. All authors contributed to the article and approved the submitted version.

## Footnotes

^1^ https://github.com/kwikteam/klustakwik2/

^2^ http://www.chronux.org

^3^ http://lantoolbox.wikispaces.com/
