# Supplementary material for: Dominance hierarchy regulates social behavior during spatial movement
Source: Front Neurosci. 2024 Feb 7;18:1237748. doi: 10.3389/fnins.2024.1237748 (PMC10879816; doi:10.3389/fnins.2024.1237748)
Supplement: Supplementary file 4 [file Data_Sheet_1.pdf]

Supplementary Information

Dominance Hierarchy regulates Social Behavior during Spatial Movement

Ariel Lara-Vasquez[1], Nelson Espinosa[1], Cristian Morales[1], Constanza Moran[1], Pablo Billeke[2], Joseph Gallagher[3], Joshua J. Strohl[3,4], Patricio T. Huerta[3,4], Pablo Fuentealba[1,5, *]

[1]Centro Integrativo de Neurociencias y Departamento de Psiquiatría, Pontificia Universidad Católica de Chile, Santiago, Chile, [2]Laboratorio de Neurociencia Social y Neuromodulación, Centro de Investigación en Complejidad Social, Universidad del Desarrollo, Santiago, Chile, [3]Laboratory of Immune & Neural Networks, Feinstein Institutes for Medical Research, Manhasset, NY, United States,[4]Department of Molecular Medicine, Zucker School of Medicine at Hofstra/Northwell, Manhasset, NY, United States, [5]Centro de Investigación en Nanotecnología y Materiales Avanzados – CIEN-UC,Pontificia Universidad Católica de Chile, Santiago, Chile

Correspondence: pjfuentealba@gmail.com

Supplementary Figures

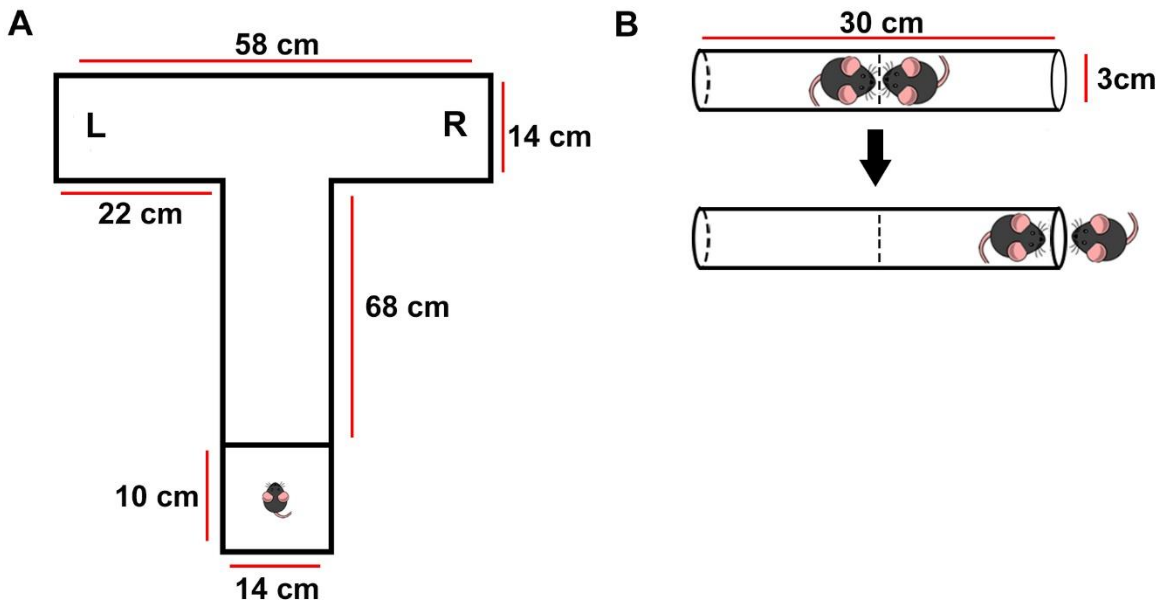

Figure S1. Behavioral apparatuses (A) T-maze (B) tube test.

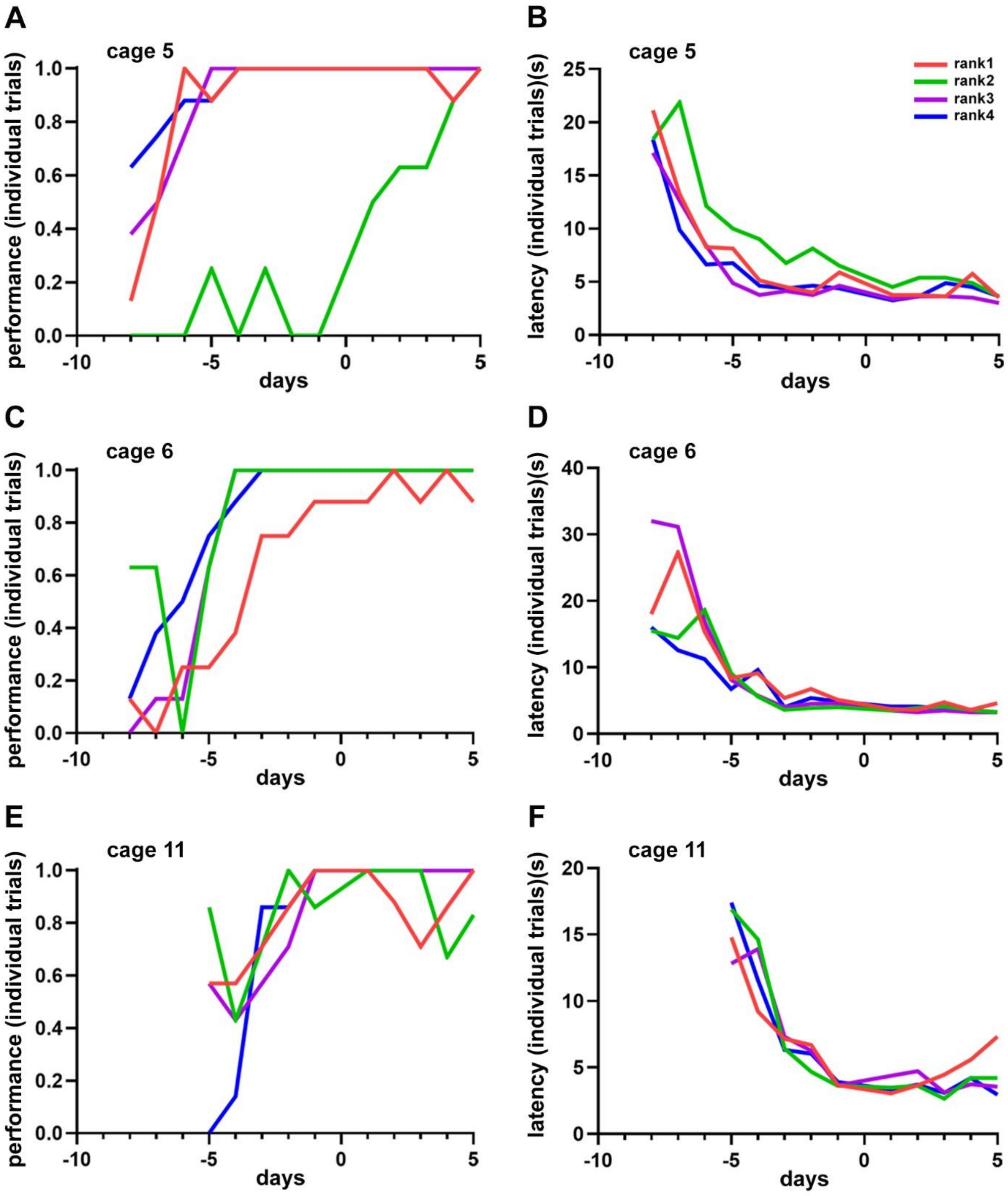

Figure S2. Example learning curves showing performance (A, C, E) and latency (B, D, F) for cages of littermates sorted by social ranking (color coded). Colored lines, average trials. Day 0 is a reference to separate individual (negative values) from collective (positive values) trials.

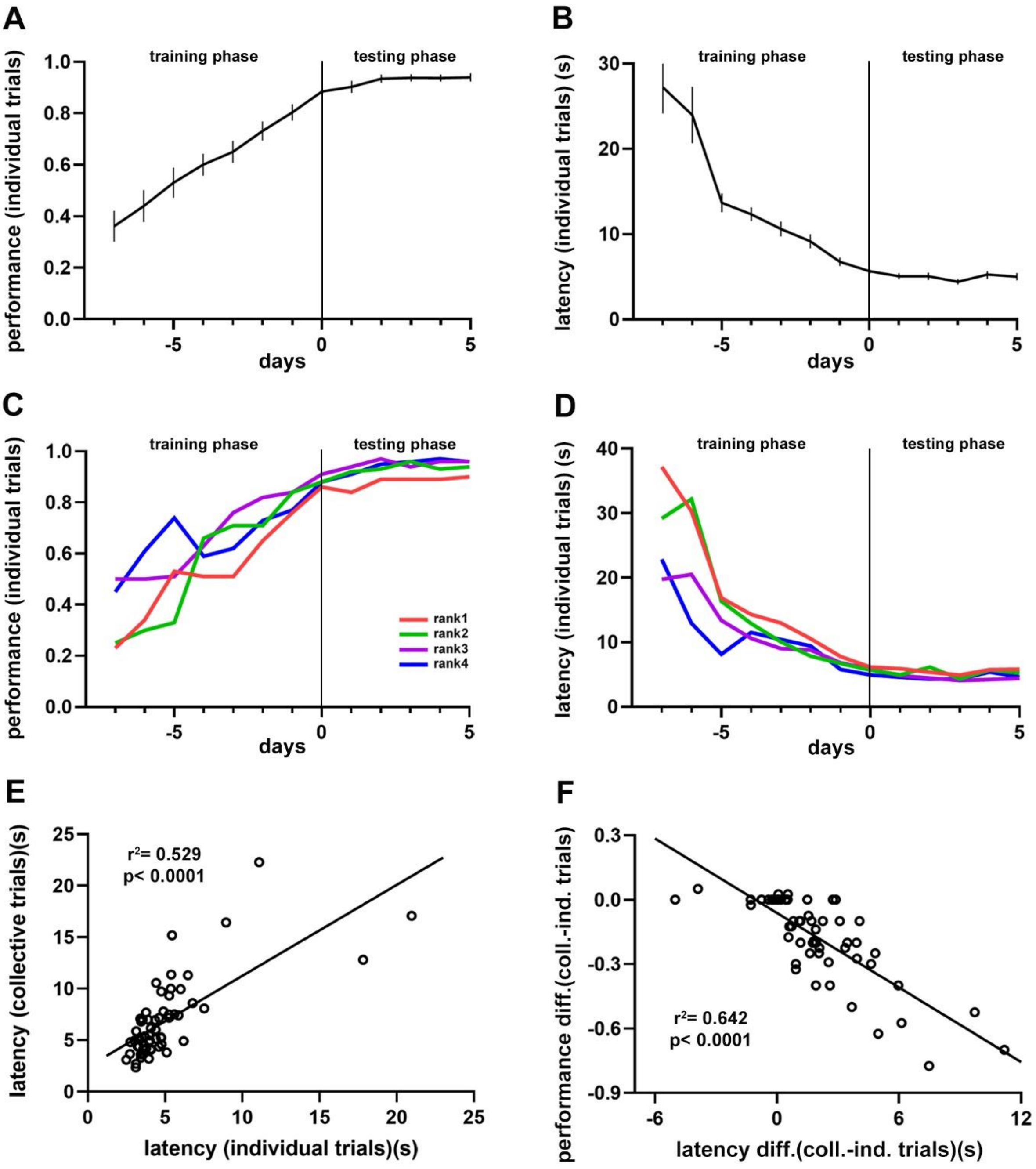

Figure S3. Average performance (A) and latency (B) during training and testing phases for all tested animals (n = 60). Data are presented as mean ± SEM. Average performance (C) and latency (D) during both training and testing phases for all tested animals sorted by social ranking (color coded) and there were no significant differences (Kruskal-Wallis test, P corrected with FDR in performance and latency, P > 0.05). E, average latency from individual trials against average latency from collective trials. F, average latency difference (collective–individual trials) against average performance difference (collective–individual trials). Black lines, population averages ± SEM; colored lines, average population.

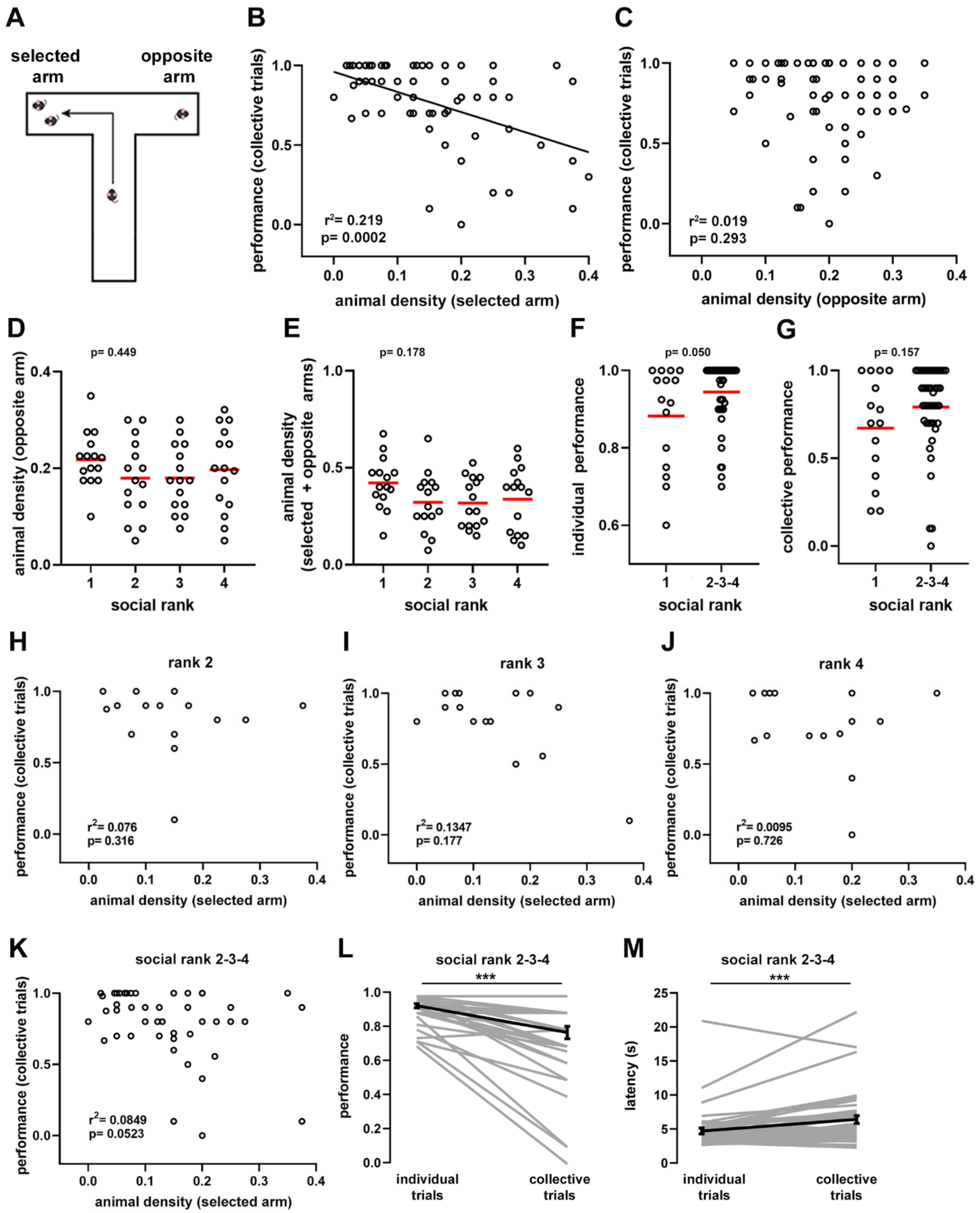

Figure S4. Density of animals in the lateral arms during collective navigation. A, Maze arms during navigation were classified as either selected (where the animal moved into) or opposite (to the selected arm). B, scatter plot of average animal density in the selected arm and performance of individual mice during collective trials (n = 60). C, scatter plot of average animal density in the opposite arm and performance of individual mice during collective trials (n = 60). D, density of animals located in the opposite arm according to social ranking. One-way ANOVA, P = 0.4499. E,

average density of animals located in both lateral arms when the choosing mouse was located at the junction according to social ranking. One-way ANOVA, P = 0.1783. F, individual task performance compared between dominant and all subordinate mice (Mann-Whitney U test, P = 0.05). G, collective task performance compared between dominant and all subordinate mice (Mann-Whitney U test, P = 0.157). H-J, scatter plot of average animal density in the selected arm and performance of individual mice during collective trials across social ranks. Circles, individual mice average; red line; population average. K, scatter plot of average animal density in the selected arm and performance of individual mice during collective trials across social rank 2, 3, and 4 (n = 45). Average task performance (L) and latency (M) for individual mice during collective and individual trials sampled during the testing phase across social ranks 2, 3, and 4 (n = 45).

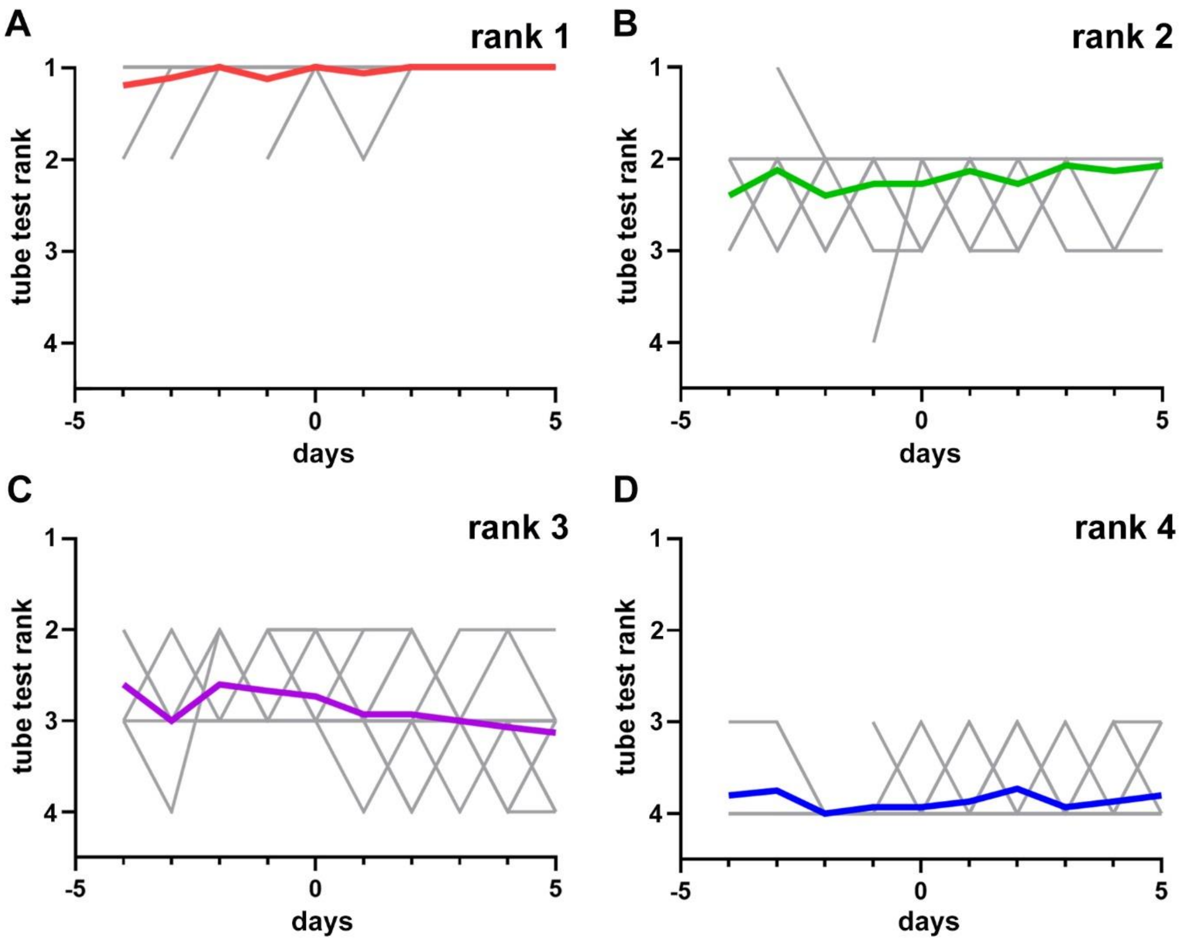

Figure S5. Dominance hierarchy of animals performing the spatial navigation task. Summary plots for all measured animals (n = 60) according to social ranking. Days -5 to 0, training phase; days 1 to 5, testing phase. A, ranking 1, dominant; B, ranking 2, first active subordinate; C, ranking 3, second active subordinate; D, ranking 4, submissive. Colored lines, population average; gray lines, individual mice. Note ranking stability over time, particularly for dominant mice.

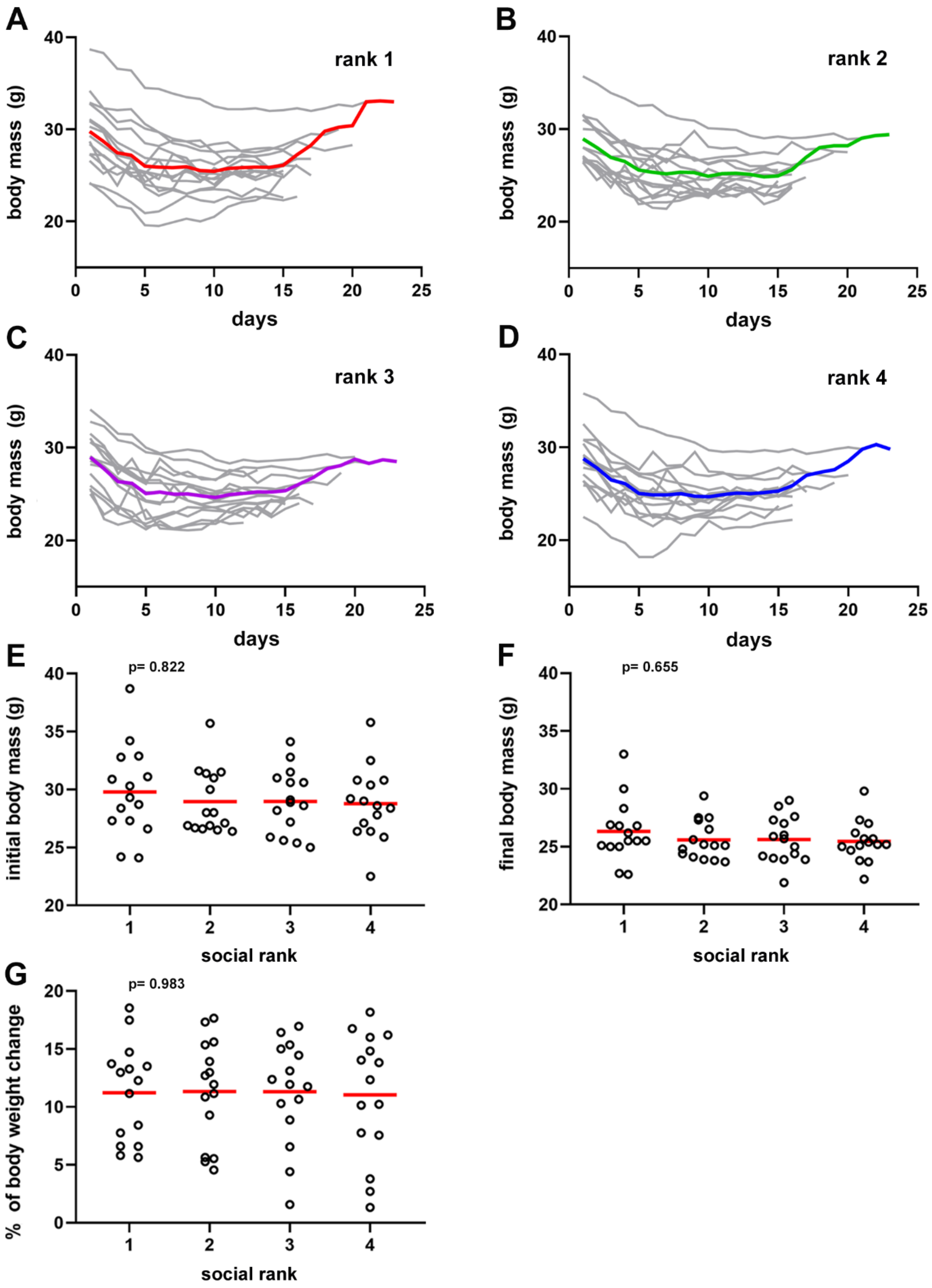

Figure S6. Body weight of animals performing the spatial navigation task. Day 0 represents initial weight. A, ranking 1, dominant; B, ranking 2, first active subordinate; C, ranking 3, second active subordinate; D, ranking 4, submissive. Food was restricted from day 1 for every mouse until it reached roughly 85% of its original weight. Summary plots for all tested animals (n = 60) according to social ranking with free access to food (E) and during food restriction (F). Note that dominant animals do not exhibit different body mass when compared to the other social rankings. G, maximal weight loss during food restriction protocol. E, One-way ANOVA, P = 0.8216; F, One-way ANOVA, P

= 0.6554. G, One-way ANOVA, P = 0.983. Colored lines, average population; gray lines, individual mice; circles, average of individual animals; red lines; population average.

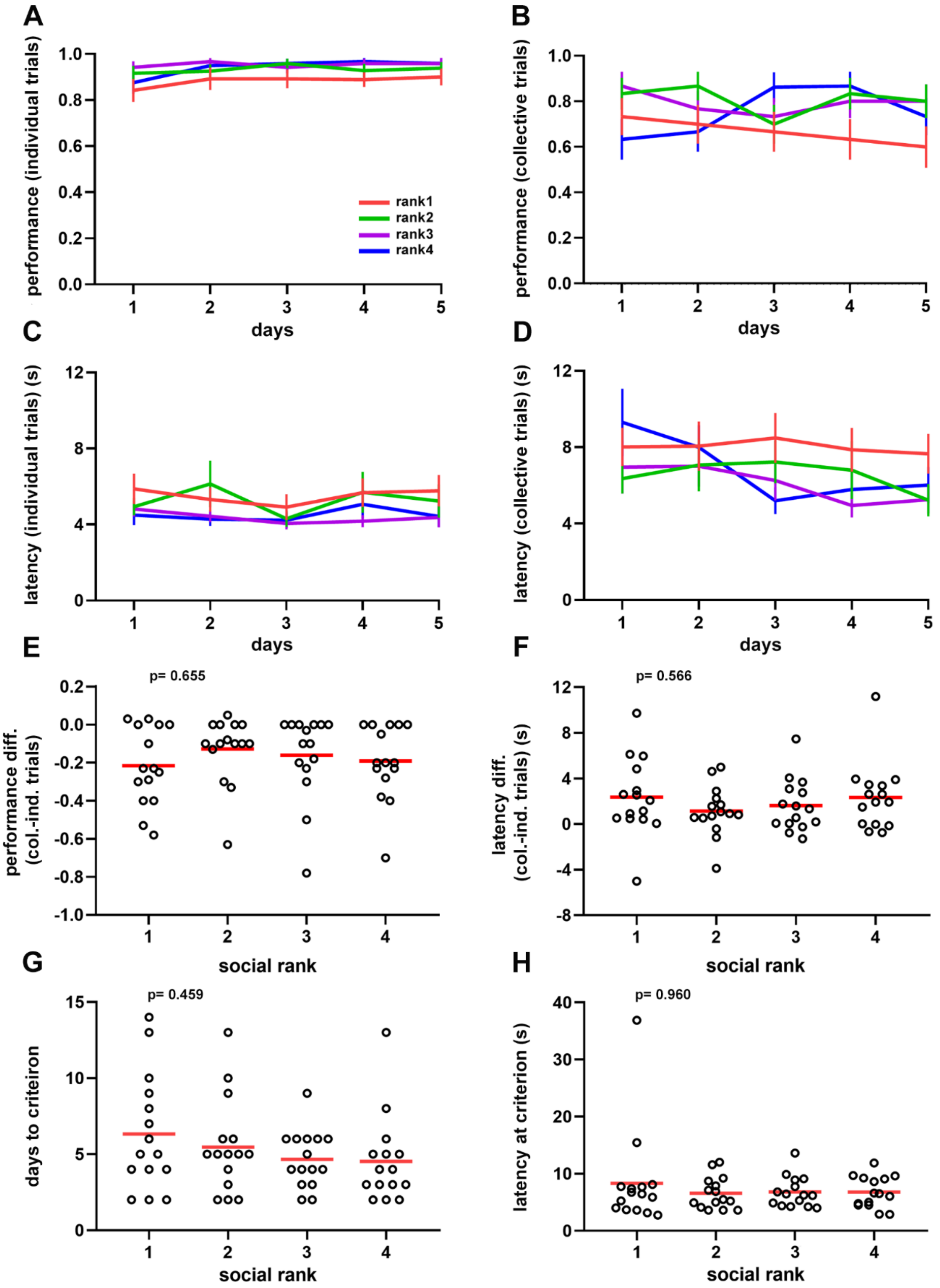

Figure S7. Average task performance (A, B) and latency (C, D) for individual (A, C) and collective (B, D) trials for all mice (n = 60) according to social ranking. Performance (E) and latency (F) difference between collective and individual trials during the testing phase of the task according to social ranking. One-way ANOVA, E, P = 0.655 F, P = 0.566. Time to reach learning criterion (G) and task latency at learning criterion (H) during the testing phase of the task according to social ranking. Kruskal-Wallis test, G, P = 0.4585; H, P = 0.9609. Colored lines, average population ± SEM; circles, average of individual animals; red lines; population average.

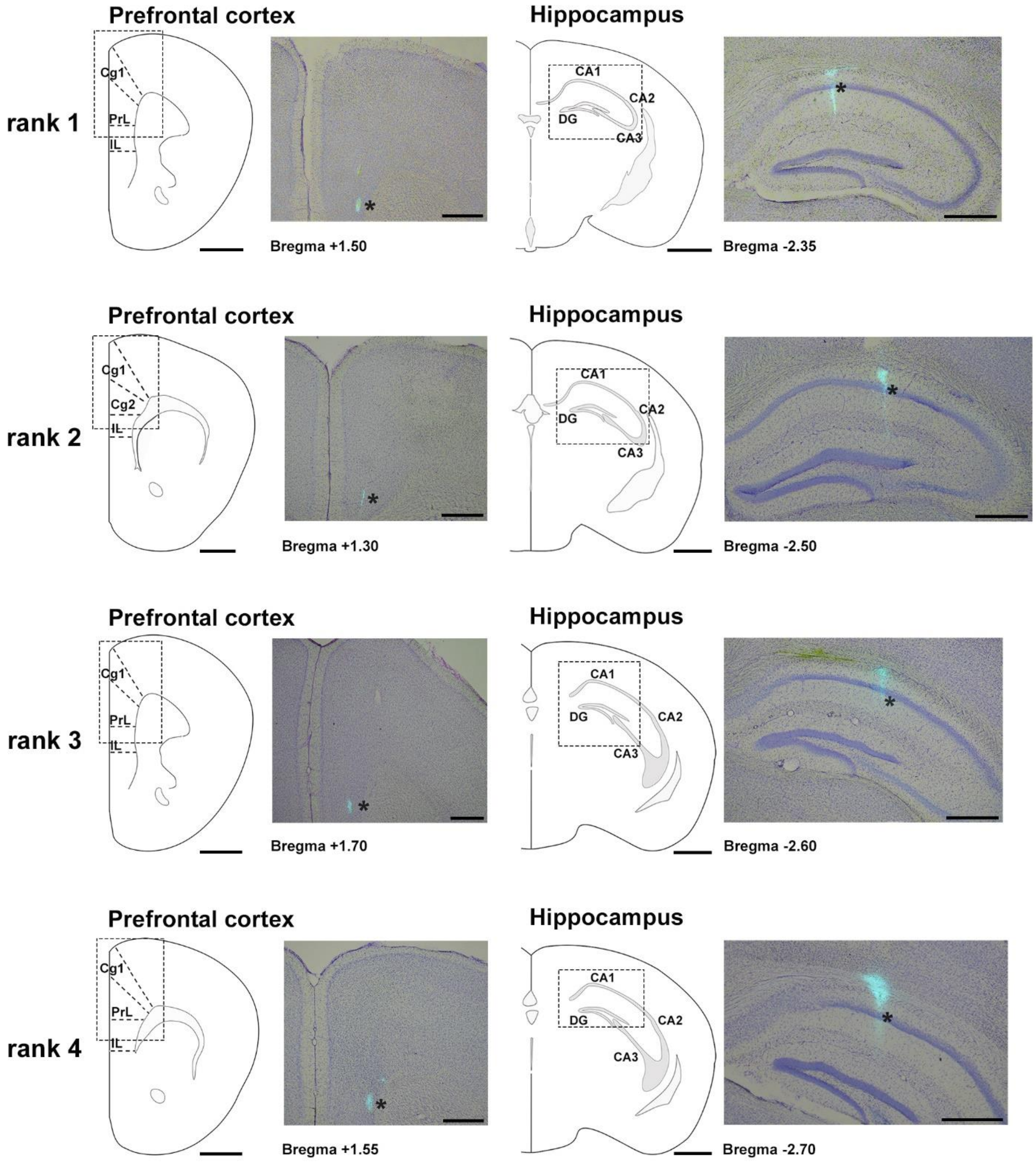

Figure S8. Anatomical location of recording electrodes. Examples sorted by social ranking. Ranking 1 (mouse CM99), ranking 2 (mouse CM65), ranking 3 (mouse CM64), ranking 4 (mouse CM47). Brain sections were Nissl stained and superimposed to fluorescent micrographs showing electrode tracks (blue, asterisks) in both cortex and hippocampus. Cg1, cingulate cortex; PrL, prelimbic cortex; IL, infralimbic cortex. CA1, CA2, CA3, cornu ammonis fields; DG, dentate gyrus. Scale bar 500 um.

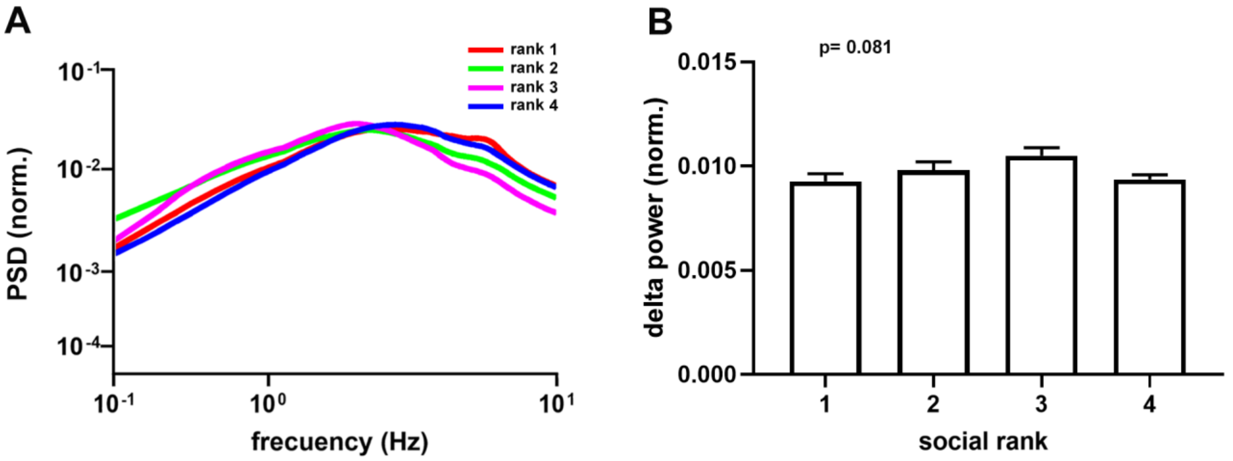

Figure S9. Cortical delta waves in anesthetized mice. Average (A) and peak (B) power spectral density of the prefrontal cortex of anesthetized mice according to social ranking (n = 22). One-way ANOVA, P = 0.081. Colored lines, averages; circles, record values; red lines; population average.

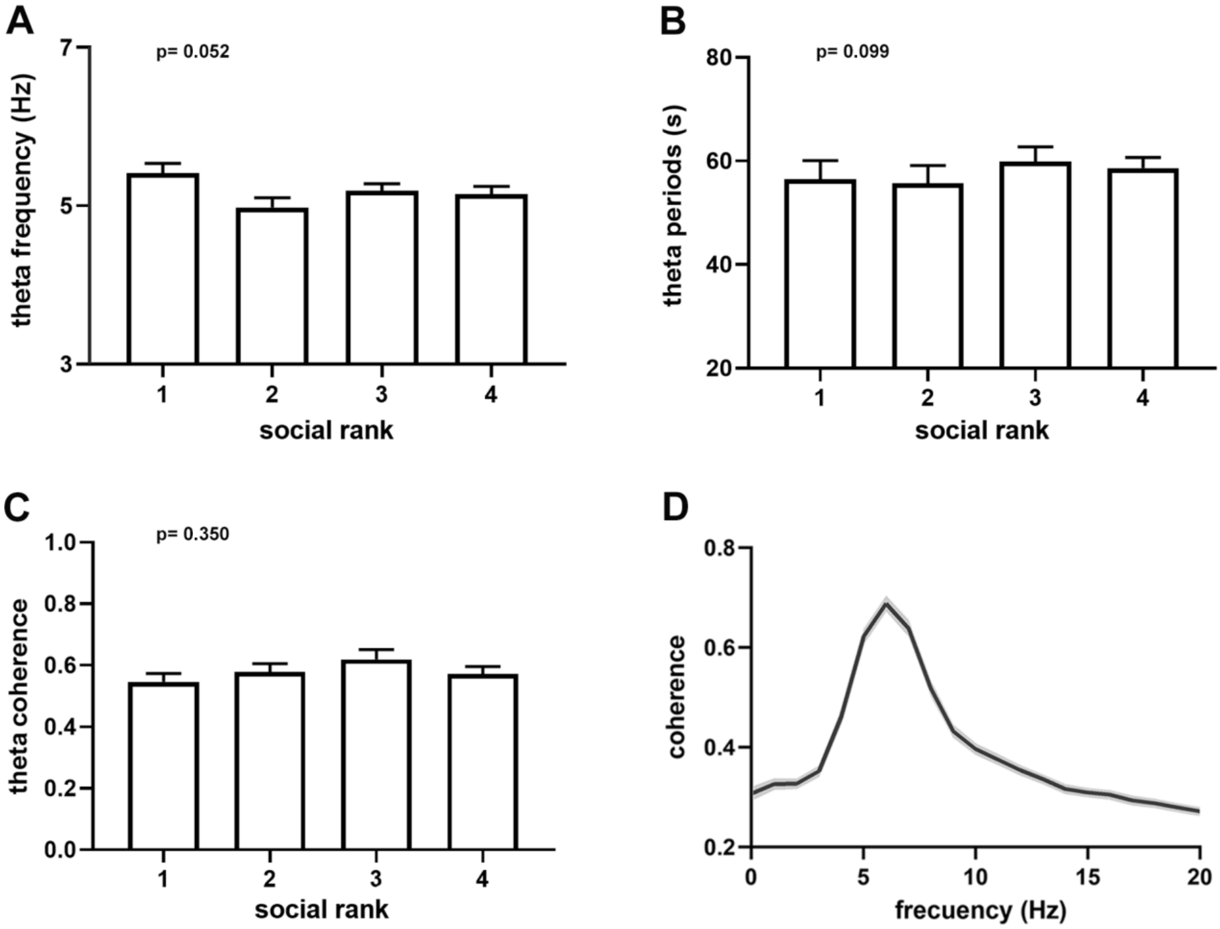

Figure S10. Theta oscillatory activity (4–8 Hz) in cortical networks. Peak frequency of theta oscillations (A) and cumulative duration of theta episodes (B) according to social ranking. One-way ANOVA; A, P = 0.0524; B, P = 0.0987. Average hippocampo-cortical spectral coherence (n = 22 animals). C, Peak hippocampo-cortical spectral coherence sorted by social ranking. One-way ANOVA, P = 0.350. D, average hippocampo-cortical spectral coherence from all recorded animals (n = 22). Data are presented as mean ± SEM. Note peak coherence at theta frequency.

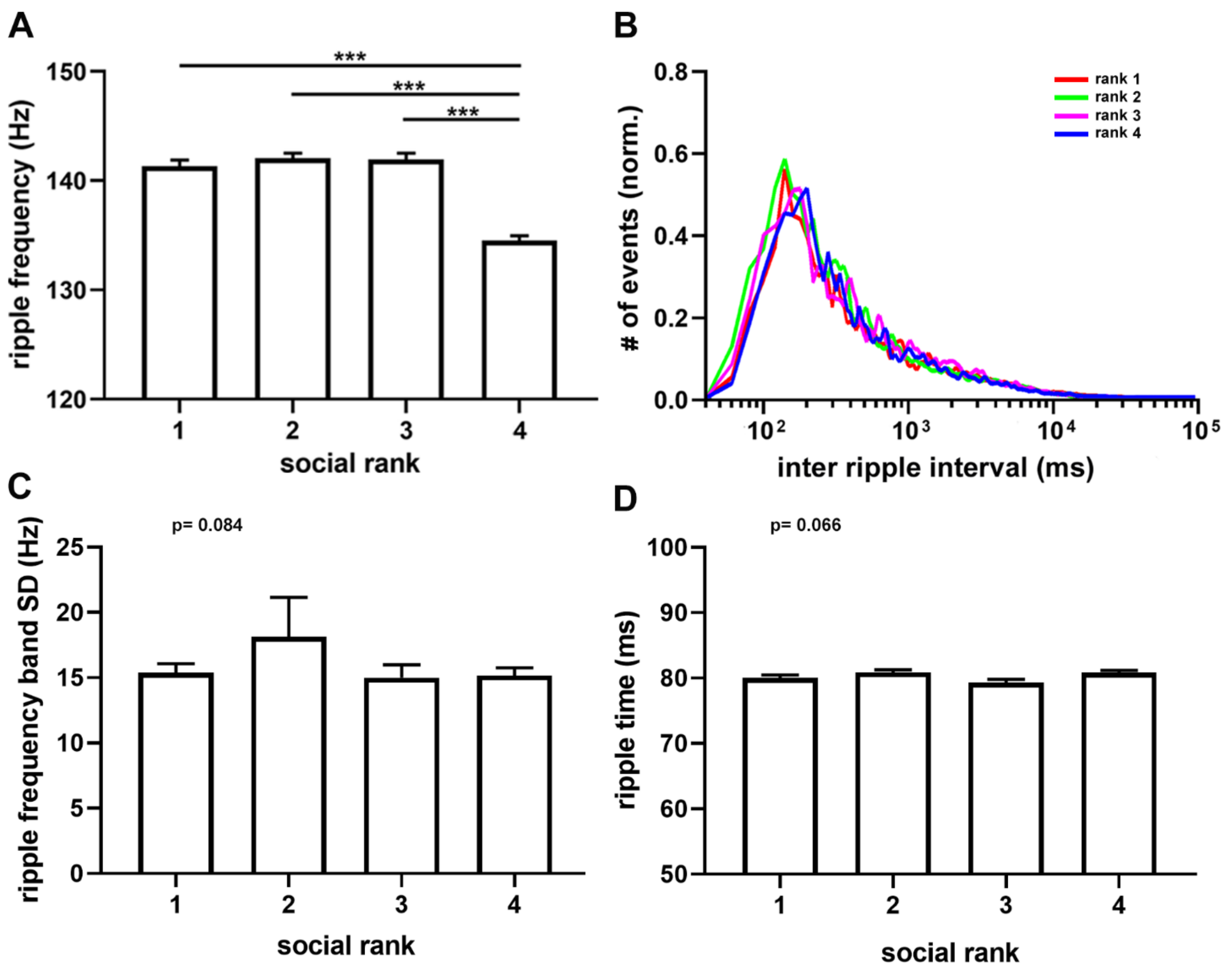

Figure S11. A, the frequency of SWRs was slower in the submissive group. One-way ANOVA, P = 5.45e-38 (F = 59.06, df =3). B, inter-event interval histogram for sharp wave ripples according to social ranking. One-way ANOVA, P = 0.619. C, variability of ripples (LFP filtered 100-250 Hz) according to social ranking. One-way ANOVA, P = 0.084. D, ripple duration in milliseconds according to social ranking. One-way ANOVA, P = 0.066

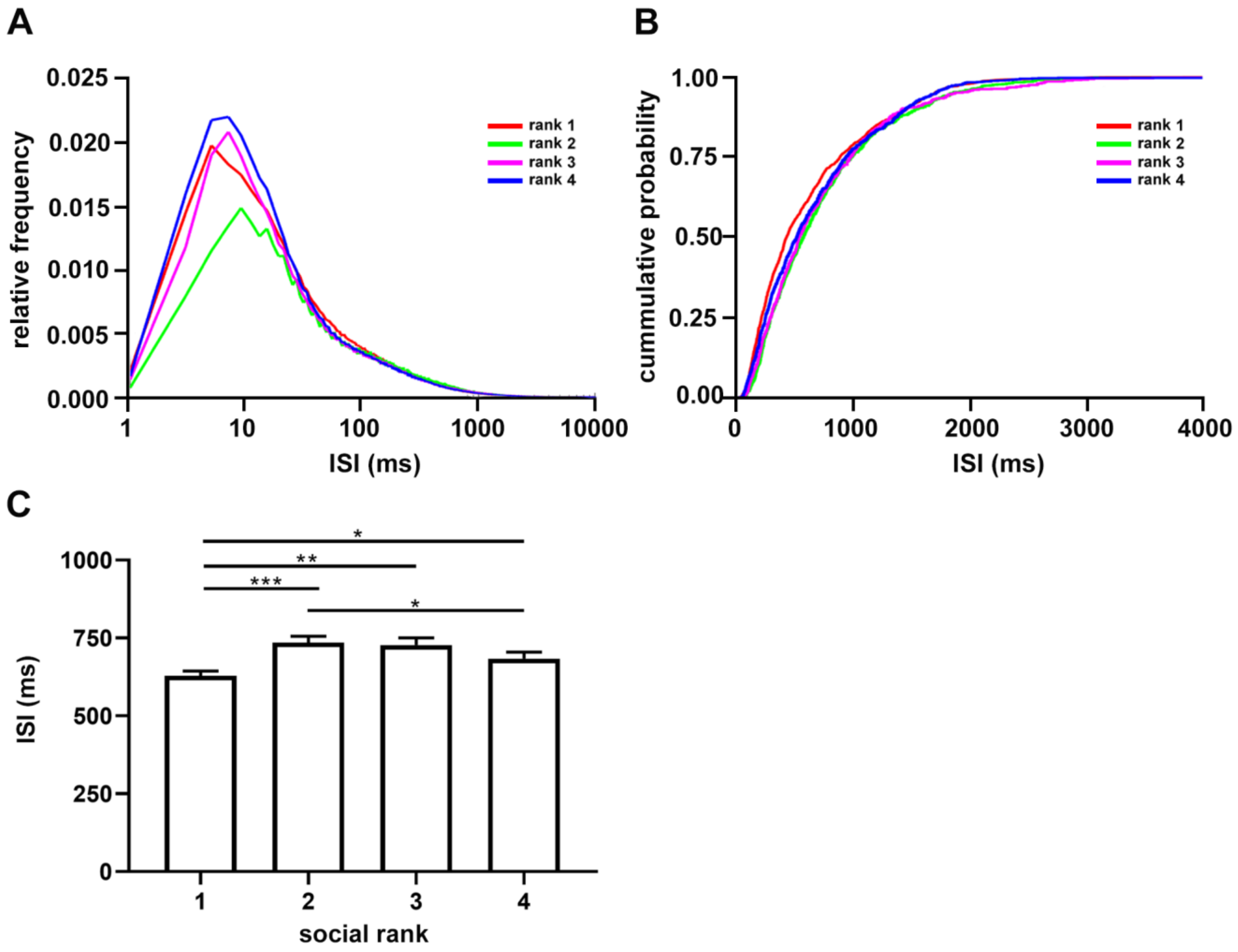

Figure S12. A, relative frequency of inter-spike intervals according to social ranking. B, cumulative probability of inter-spike intervals according to social ranking. C, comparison of inter-spike intervals according to social ranking (Kruskal-Wallis test, P = 4.94e-08, Chi-sq =36.85. dg =3). Dominant mice exhibit shorter inter-spike intervals, consequent with faster discharge rates.

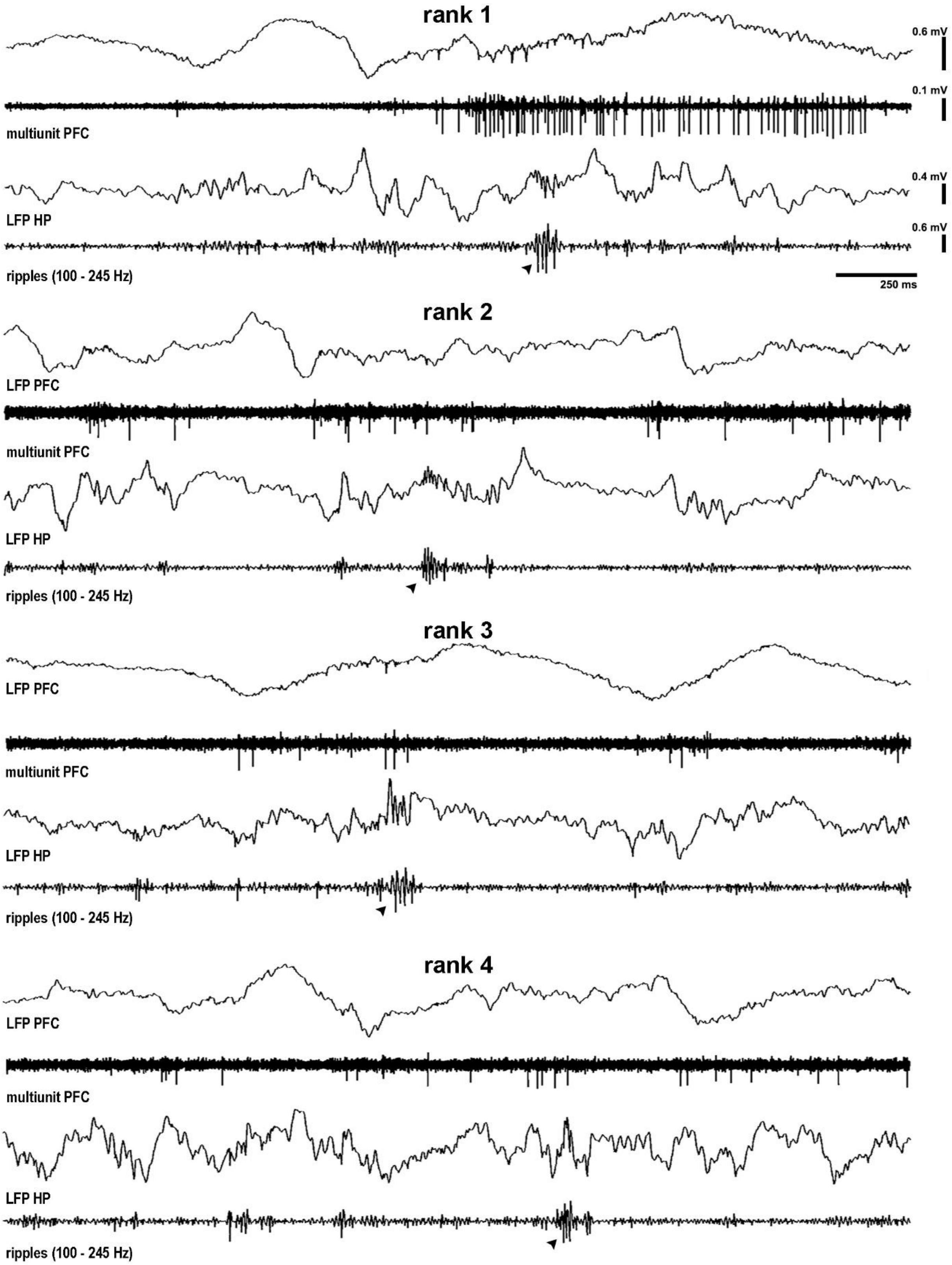

Figure S13. Examples of hippocampal sharp wave ripples and associated prefrontal cortex spiking for all social groups. Simultaneous recordings of prefrontal cortex (LFP PFC) and hippocampus (LFP HP) showing sharp wave ripples (ripples, filtered 100-250 Hz, arrowhead) and cortical spiking activity (multiunit PFC, filtered 300-4000 Hz) recorded in urethane-anesthetized mice: Rank 1, mouse CM24_reg05; rank 2, mouse CM73_reg02; rank 3, mouse CM28_reg02; rank 4, mouse CM98_reg01.

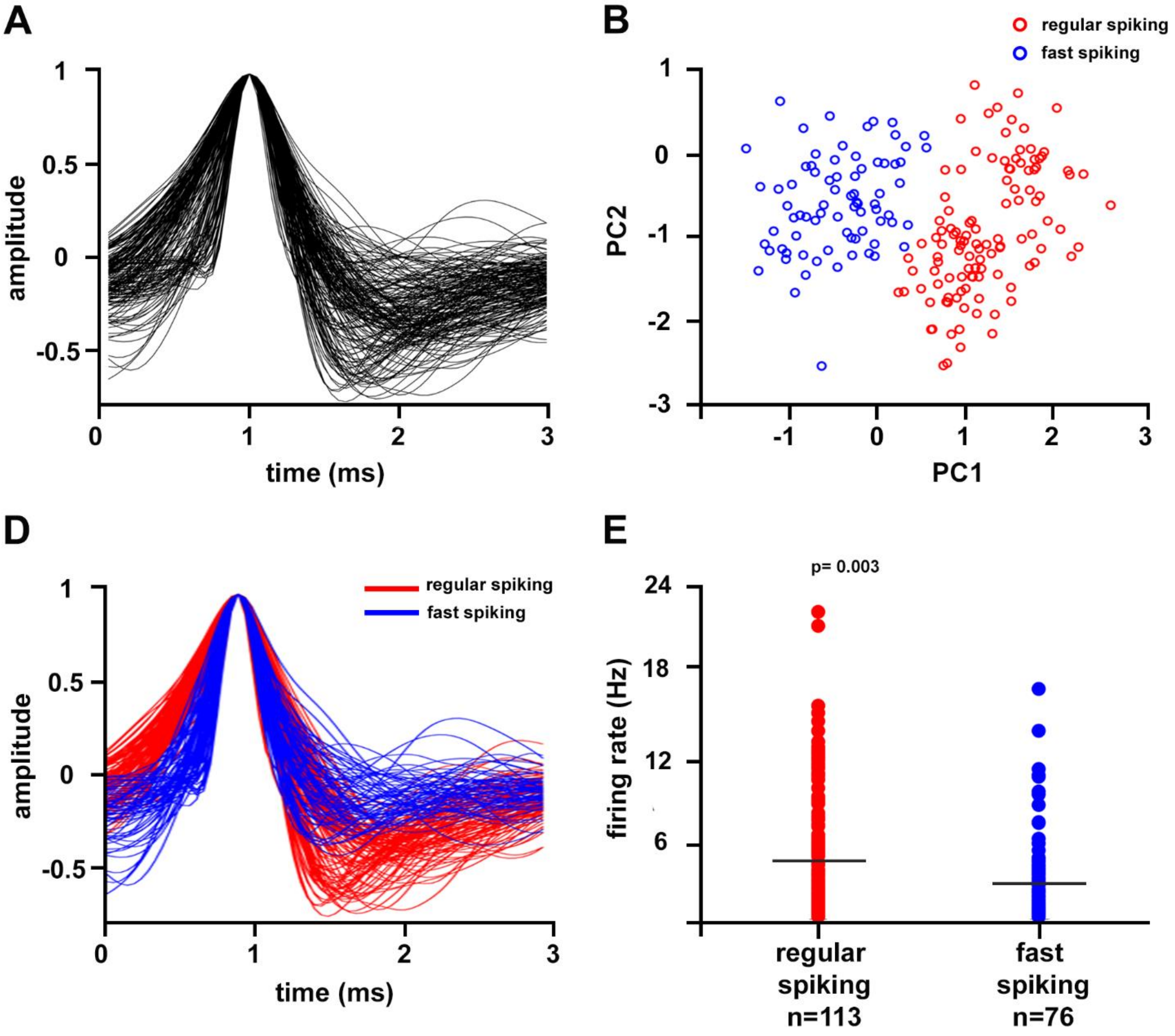

Figure S14. Single units identified in freely-moving recordings. A, Overlay of all average waveforms detected (n = 189). B, Scatter plot of the two principal components (PC) for every cluster (unit) identified). Note clusters are not clearly segregated. D, Overlay of average waveforms classified as either putative interneurons (fast spiking) or putative pyramidal cells (regular spiking). E, comparison of firing rates between putative interneurons and putative pyramidal cells (One-sample t-test, n=189). Note that putative pyramidal cells exhibit higher firing rates than putative interneurons.

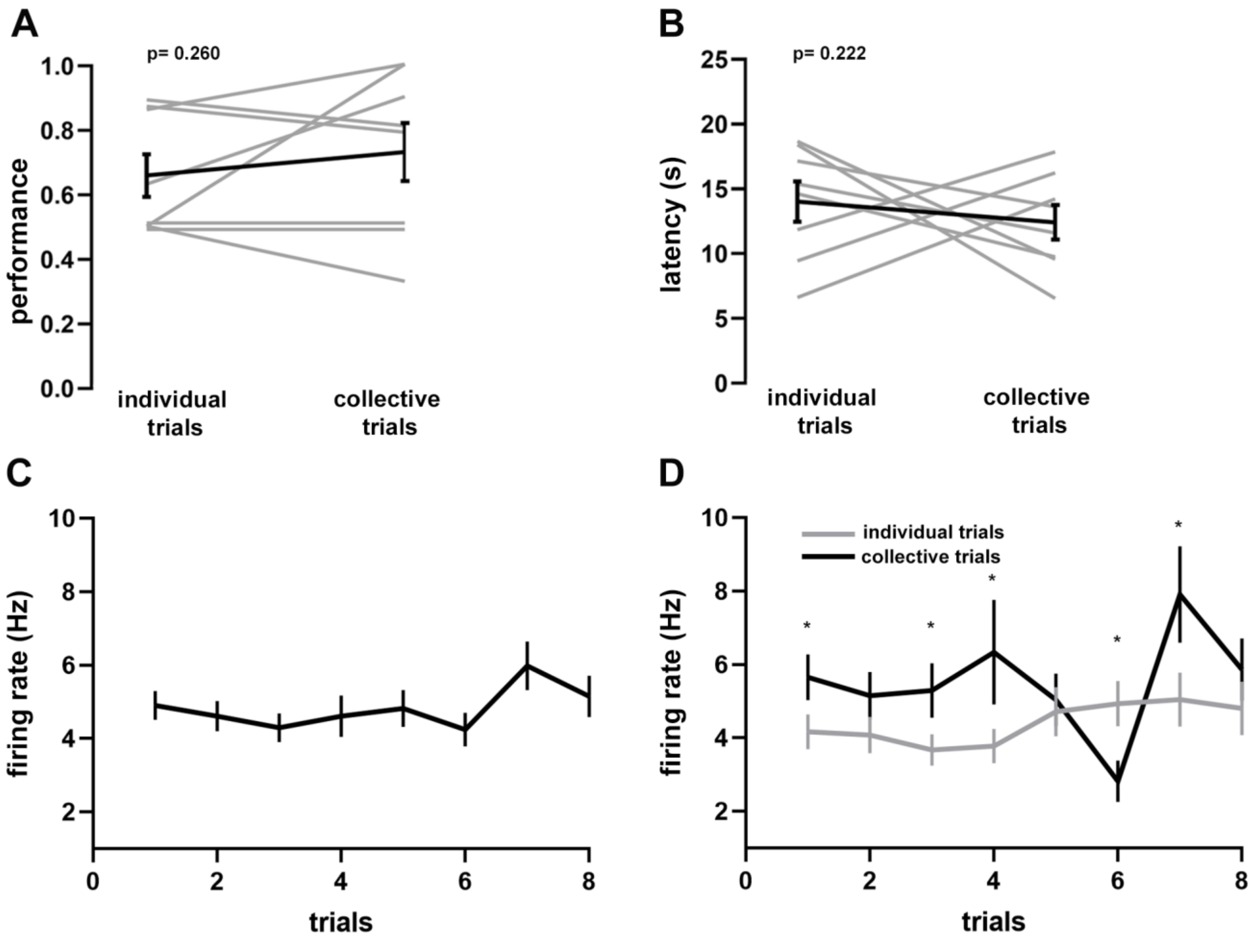

Figure S15. Average task performance (A) and latency (B) for chronically-implanted mice during collective and individual trials sampled during collective navigation (n = 8). One-sample t-test; A, P = 0.2597; B, P = 0.2217. C, average firing rate from all recorded units (n = 189) from chronically-implanted mice during task performance across trials. D, average firing rate from cortical units sorted by social condition and trial number. Population averages ± SEM; bars, average ± SEM. Asterisks depict significant differences (Mann-Whitney U test, P < 0.05).

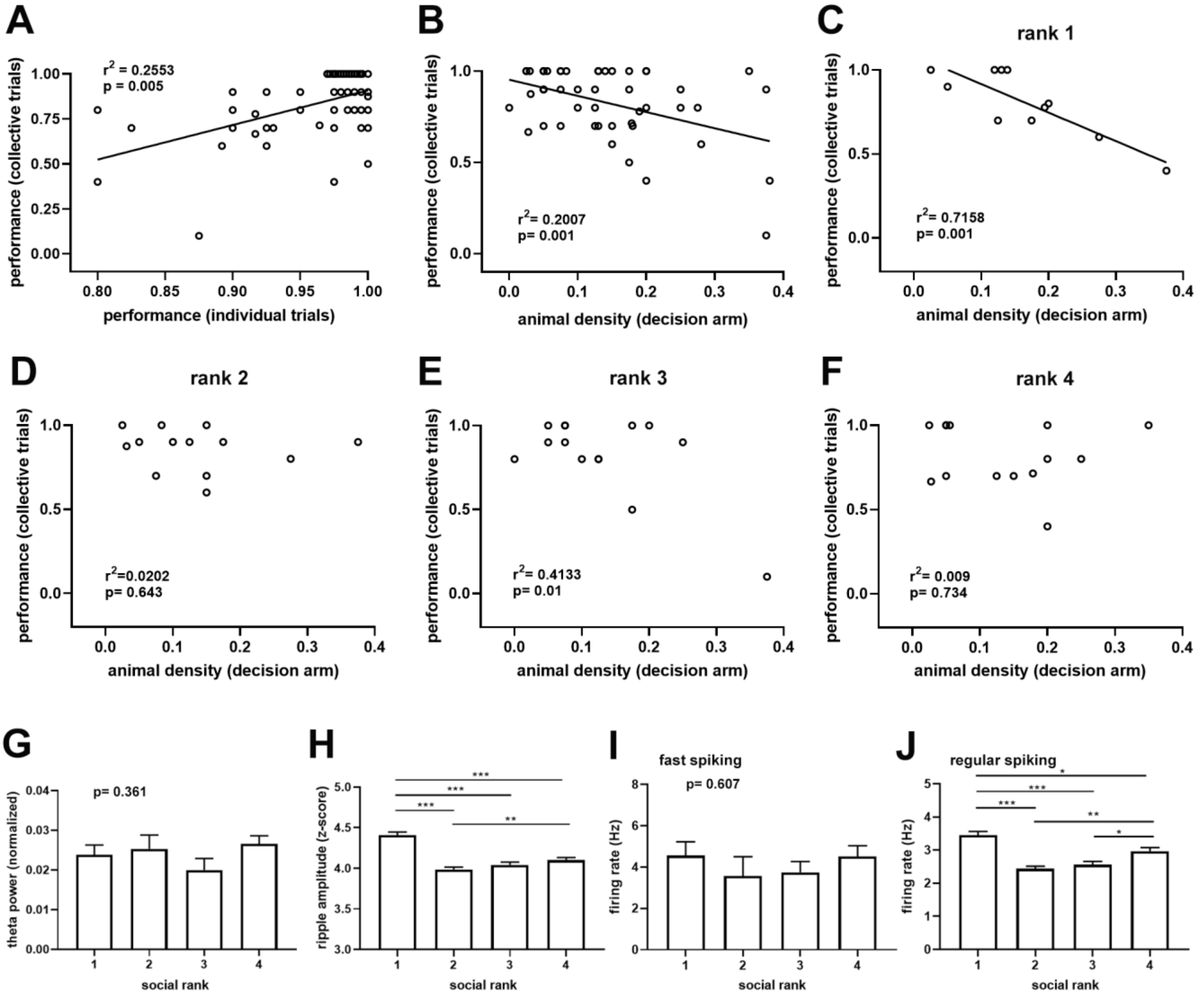

Figure S16. Behavioural and electrophysiological parameters across social rankings. Changing the learning criterion from 75% to 80% success rate did not affect results. A, linear regression between individual and collective performance. B, scatter plot of task performance versus animal density in the decision arm for all animals. C-F, scatter plot of task performance versus animal density in the decision arm sorted by social ranking. G, theta power sorted by social ranking. H, ripple amplitude sorted by social ranking (One-way ANOVA, $P = 6.12e-17$, $F = 26.26$, dg = 3). I, firing rate of putative interneurons sorted by social ranking. J, firing rate of putative pyramidal cell sorted by social ranking (One-way ANOVA, $P = 5.98e-14$, $F = 21.78$, dg = 3). Asterisks depict significant differences ($P < 0.05$).

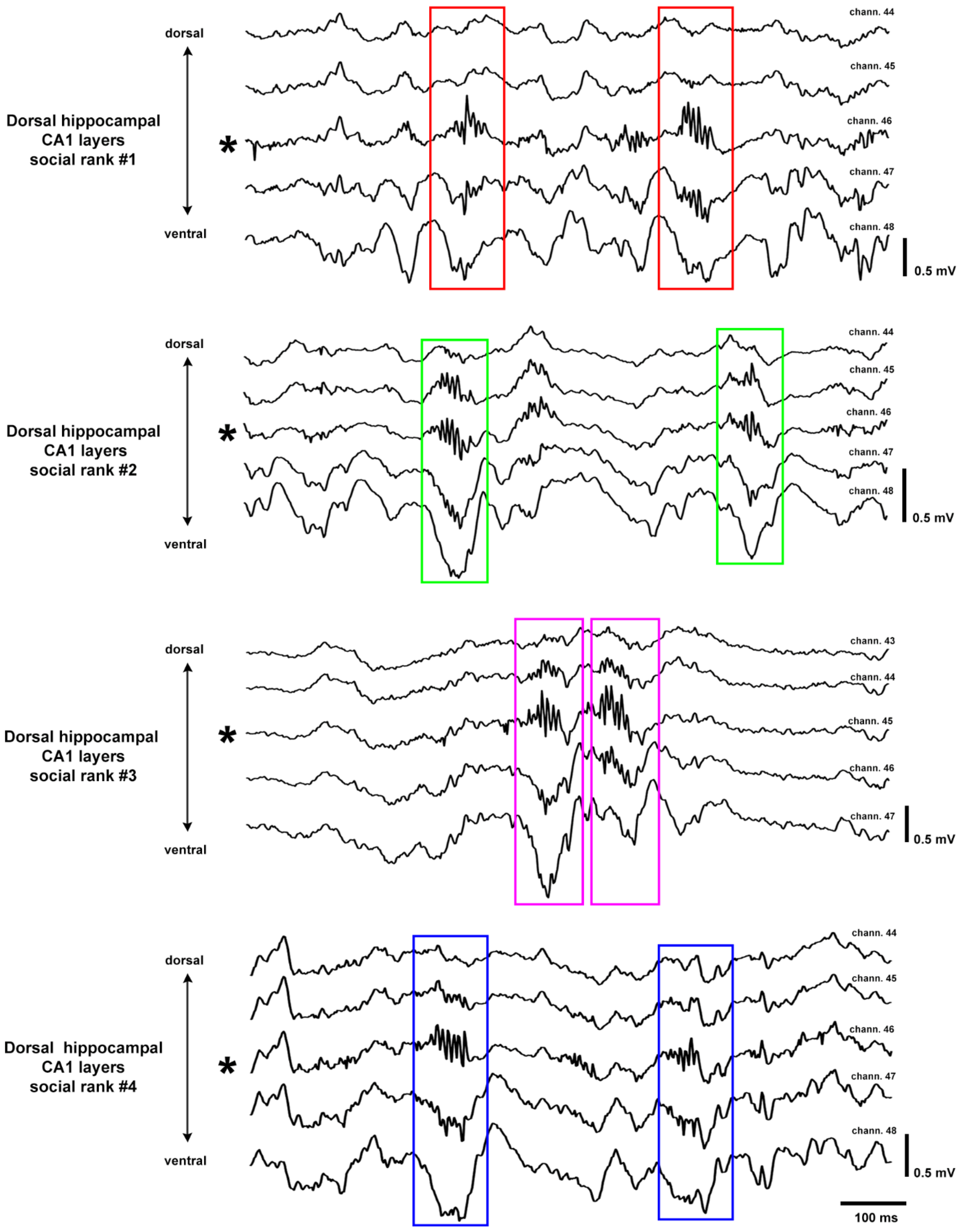

Figure S17. Depth profile recordings of sharp wave ripples across the dorsal CA1. Example recordings with linear multielectrodes (separation 100 µm) from individuals from every social ranking. Asterisks depict in each case the channel selected for ripple analysis.

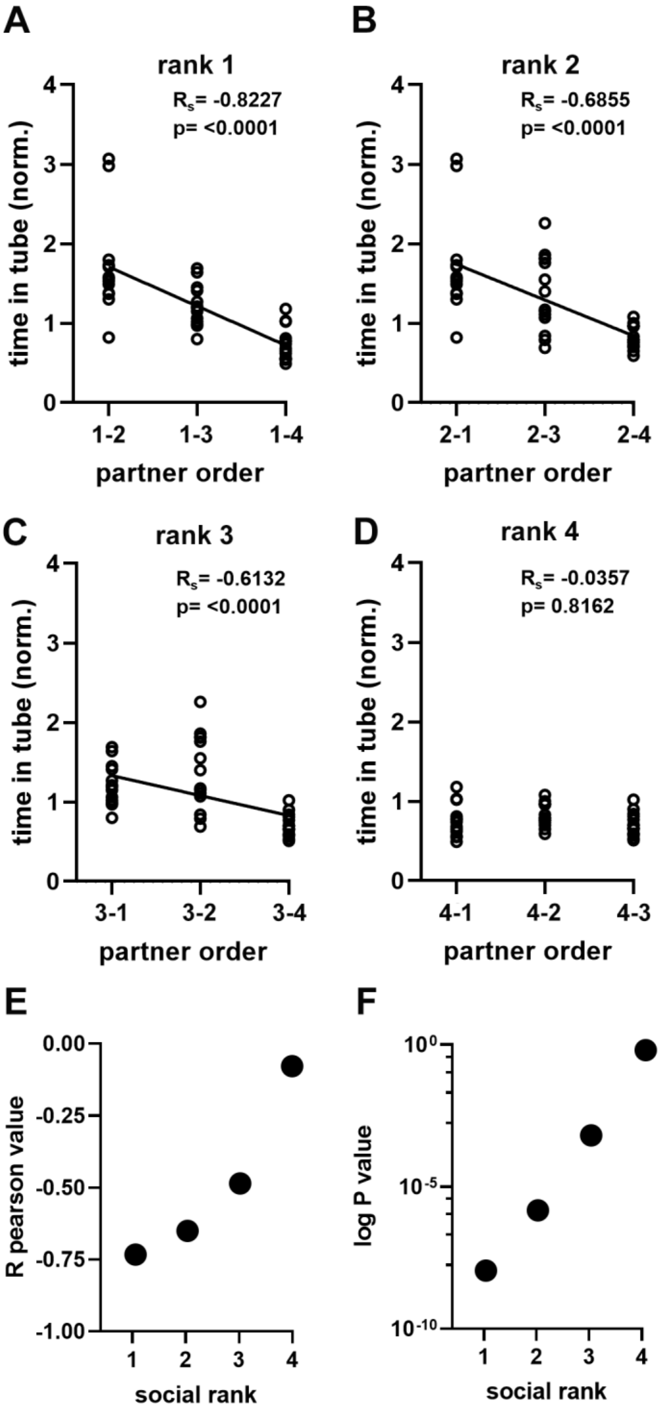

Figure S18. Time spent in the tube test for each animal against littermates. A-D, Normalized time spent in the tube test plotted against three pairing conditions for each social rank. E, Spearman coefficient (data obtained in A, B, C, and D) plotted against social rank. F, p-value (data obtained in A, B, C, and D) plotted against social rank. Rs, Spearman coefficient, 1–2 stands for rank-1 against rank-2 and so on; N=60.

Supplementary tables

Table S1. Mixed logistic model with fixed effects for collective task performance (n = 60 animals used for behavioral tests)

**Parameter**	**estimate**	**SE**	**P**
intercept	0.2721	0.8258	0.741770
individual performance	2.8035	0.8182	*0.000612*
animal density (selected arm)	-3.1858	0.4876	*6.41e-11*
animal density (opposite arm)	-1.5323	0.4978	*0.002085*
total animal density	1.0492	0.3206	*0.001066*

Table S2. Mixed logistic model with fixed effects for collective task performance (n = 20 animals used for anesthesia recordings)

**Parameter**	**estimate**	**SE**	**P**
intercept	-0.1794	1.3608	0.89513
individual performance	3.1845	1.3582	*0.01905*
animal density (selected arm)	-2.4680	-3.075	*0.00211*
animal density (opposite arm)	-1.1839	0.7862	0.13
total animal density	0.78	0.52	0.13

Table S3. Univariate tests of significance for firing rate (n = 20 animals used for anesthesia recordings)

**parameter**	**SS**	**F**	**P**
intercept	10187.48	1169.875	*10e-6*
cortical region	15.66	1.799	0.179932
neuron type	325.64	37.395	*10e-6*
hierarchy	93.78	3.590	*0.013127*
cortical region*neuron type	20.00	2.297	0.129738
cortical region*hierarchy	64.68	2.476	0.059632
neuron type*hierarchy	40.71	1.558	0.197450
cortical region*neuron type*hierarchy	64.79	2.480	0.059302

SS, sum of squares; F, F-statistic; P, p-value; cortical region (dorsal or ventral prefrontal cortex); neuron type (fast spiking or regular spiking cells); hierarchy (dominant, first active subordinate, second active subordinate, or submissive).

Table S4. Animals used for behavioural and electrophysiological experiments.

# of animal	ID	cage	social rank	record anesthesia ID	# of neurons (anesthesia)	# of neurons (freely-moving, individual trials)	# of neurons (freely-moving, collective trials)
1	ti1088	234A	1				
2	td1091	234A	2				
3	cn1090	234A	4				
4	cm1089	234A	3				
5	ti1258	240A	1				
6	td1257	240A	2				
7	cm1255	240A	4				
8	cn1259	240A	3				
9	cm1314	247A	3				
10	ti1311	247A	1				
11	cn1313	247A	4				
12	td1312	247A	2				
13	cn1426	268A	2				
14	cm1429	268A	4				
15	td1428	268A	1				
16	ti1427	268A	3				
17	cn1433	270A	3				
18	cm1430	270A	1				
19	td1432	270A	4				
20	ti1431	270A	2				
21	cm1499	276A	3	CM16	188		
22	cn1498	276A	1				
23	td1501	276A	2	CM17	218		
24	ti1500	276A	4				
25	cm1506	280A	1	CM24	317		
26	cn1503	280A	4	CM23	74		
27	td1504	280A	3	died in surgery			
28	ti1505	280A	2	CM22	110		
29	cm1508	284A	1	CM29	135		
30	cn1510	284A	4	CM26	78		
31	td1507	284A	3	CM28	121		
32	ti1509	284A	2	CM27	17		
33	cm1674	312A	4				
34	cn1671	312A	1				
35	td1672	312A	2				
36	ti1673	312A	3				
37	cm1676	313A	3				
38	cn1675	313A	1				

# of animal	ID	cage	social rank	record anesthesia ID	# of neurons (anesthesia)	# of neurons (freely-moving, individual trials)	# of neurons (freely-moving, collective trials)
39	td1678	313A	2				
40	ti1677	313A	4				
41	cm1759	328A	1	CM48	58		
42	cn1756	328A	4	CM47	190		
43	td1758	328A	3	CM46	206		
44	ti1757	328A	2	died in surgery			
45	cm1846	339A	3	CM64	47		
46	cn1847	339A	1	CM67	153		
47	ti1845	339A	2	CM65	180		
48	ti1844	339A	4	CM66	193		
49	cm1850	340A	4	CM74	339		
50	cn1851	340A	2	CM73	283		
51	td1849	340A	3	CM72	104		
52	ti1848	340A	1	CM75	245		
53	ti1900	344A	3				
54	cn1901	344A	4	CM98	62		
55	td1903	344A	1	CM99	384		
56	cm1902	344A	2				
57	cm1912	345A	1				
58	cn1909	345A	4				
59	td1911	345A	3				
60	ti1910	345A	2				
61	H8242	H240	1			12	5
62	H8243	H240	4			9	12
63	H8252	H825	1			18	14
64	H8254	H825	4			15	17
65	H8361	H836	1			8	5
66	H8363	H836	4			9	16
67	H8372	H837	1			15	10
68	H8373	H837	4			9	15
				**Total neurons**	**3702**	**95**	**94**

Table S5. Summary of neurons recorded in electrophysiological experiments.

social rank	anesthesia neurons (n=22)	freely moving neurons (n=8)
rank 1	1292 (n=6)	87 (n=4)
rank 2	808 (n=5	
rank 3	666 (n=5)	
rank 4	936 (n=6)	102 (n=4)
	total neurons = 3702	total neurons =189

Supplementary Videos

video	file	trial	mouse	cage	description
S1	tube_test	5	ti01, ca02	212A	Two littermates in the tube test
S2	individual_acute	1	ti1844	339A	T-maze individual trial of dominant mouse
S3	collective_acute	3	ti1844, td185, cm1846, cn1847	339A	T-maze collective trial of four littermates
